# A novel stress response pathway mediates biofilm architecture in *Pseudomonas aeruginosa*

**DOI:** 10.1371/journal.ppat.1013832

**Published:** 2026-07-28

**Authors:** Ainelen Piazza, Catriona M. A. Thompson, Govind Chandra, Harshanie Dasanayaka, Gerhard Saalbach, Carlo Martins, Eleftheria Trampari, Mark A. Webber, Freya Harrison, Jacob G. Malone

**Affiliations:** 1 Department of Molecular Microbiology, John Innes Centre, Norwich Research Park, Norwich, United Kingdom; 2 The Centre for Microbial Interactions, Norwich Research Park, Norwich, United Kingdom; 3 John Innes Centre Proteomics Platform, Norwich, United Kingdom; 4 Quadram Institute Bioscience, Norwich Research Park, Norwich, United Kingdom; 5 School of Life Sciences, Gibbet Hill Campus, The University of Warwick, Coventry, United Kingdom; 6 School of Biological Sciences, University of East Anglia, Norwich Research Park, Norwich, United Kingdom; Dartmouth Medical School: Dartmouth College Geisel School of Medicine, UNITED STATES OF AMERICA

## Abstract

*Pseudomonas aeruginosa* is a multidrug-resistant opportunistic pathogen, with chronic infections often associated with biofilm formation. Here, we investigate the previously uncharacterized gene *PA3049*, which is upregulated under biofilm conditions, to determine its role in infection, biofilm formation, and antimicrobial sensitivity. We show that the small uncharacterised protein PA3049, renamed as *B*iofilm *a*rchi*t*ecture *R*egulator (BatR), promotes biofilm establishment and enhances biofilm survival in sub-inhibitory concentrations of antibiotics. Proteomic analysis revealed that BatR influences the R2/F2 pyocin cluster, which drives explosive cell lysis and extracellular DNA (eDNA) release during biofilm development. We further identify a specific interaction between BatR and PA0486 (SrkA), an uncharacterised Ser/Thr protein kinase. We show that SrkA controls biofilm and pyocyanin production, and lysis-mediated eDNA release through regulation of the R2/F2 pyocin cluster and activation of bacteriophage Pf4. Our findings support a model in which SrkA directly regulates key biofilm-associated phenotypes, while BatR acts as a modulatory partner that tunes SrkA activity under specific conditions. Finally, BatR function was tested in high-validity infection models, including the *ex vivo* pig lung model of cystic fibrosis infection and a synthetic chronic-wound model. In these models, BatR contributes to biofilm architecture and antibiotic resistance and modulates pyocyanin production. Our study implicates the BatR/SrkA system in the response of *P. aeruginosa* biofilms to antibiotic challenge in lung infections.

## Author summary

Biofilm formation by the opportunistic pathogen *Pseudomonas aeruginosa* represents a major clinical challenge, with biofilms typically associated with chronic infection, increased antibiotic resistance and infectious persistence. As part of a wider study of *P. aeruginosa* host colonisation, we discovered a small uncharacterised protein: Biofilm architecture Regulator (BatR) that markedly enhances biofilm survival when exposed to sub-inhibitory concentrations of antibiotics. BatR contributes to biofilm architecture and antibiotic resistance in clinically relevant infection models, including the *ex vivo* pig lung model of cystic fibrosis infection and a synthetic chronic-wound model. BatR directly interacts with the Ser/Thr protein kinase SrkA. Together, BatR and SrkA control pyocyanin production and shape biofilm architecture and lysis-mediated eDNA release via activation of the R2/F2 pyocin cluster and bacteriophage Pf4. We propose a model in which SrkA regulates key biofilm-associated phenotypes, while BatR acts as a regulatory partner that tunes SrkA activity under specific conditions. The BatR/SrkA system appears to play in important role in the response of *P. aeruginosa* biofilms to antibiotic challenge in a range of chronic infections.

## Introduction

*Pseudomonas aeruginosa* is a virulent, opportunistic human pathogen, designated as one of the ESKAPE group of bacteria [1] that are leading causes of multidrug-resistant, nosocomial bacterial infections. *P. aeruginosa* presents a significant challenge in healthcare settings where it causes highly persistent, chronic infections, primarily affecting catheter and cannula implant [2], burn or other major injury victims [3], chronic wounds [4], and immunocompromised patients [5]. *P. aeruginosa* is also a major respiratory pathogen, causing both acute and chronic lung infections. Pulmonary infections caused by *P. aeruginosa* are a major cause of mortality and morbidity in people with the genetic disorder cystic fibrosis (CF) [6,7].

Chronic *P. aeruginosa* infections are frequently associated with biofilms, which enable it to evade host immune responses and confer broad resistance to antimicrobial agents, complicating treatment strategies. Bacterial biofilms exhibit common traits and phenotypic characteristics, including cell-to-cell communication (quorum sensing), the production and deployment of extracellular polymeric substances and extracellular DNA (eDNA), and the spatially structured control of motility, adhesins and cyclic di-GMP (c-di-GMP) levels [8]. The upregulation of genes linked to stationary phase adaptation, environmental stress and anaerobiosis further underscores the distinct features associated with biofilm growth [9,10]. Additionally, the spatial arrangement of cells within the biofilm community, exposed to multiple resource gradients, introduces heterogeneity in cell physiology and metabolism that plays an important role in antibiotic tolerance. This diversity includes significant subpopulations of less metabolically active (dormant) cells, which contribute to the notable tolerance of biofilms to antibiotics designed to target active metabolic processes [11]. For example, dormant cells within oxygen-depleted zones of *P. aeruginosa* biofilms exhibit lower overall mRNA transcript abundance and increased tolerance to ciprofloxacin and tobramycin [11].

A crucial aspect of biofilm-associated infections is the frequent exposure to sub-inhibitory concentrations (SICs) of antibiotics, which do not kill bacteria but can still influence community behaviour [12,13]. SICs often occur in tissues where antibiotic penetration is incomplete [14], in biofilms where the matrix limits drug diffusion [15], or when antibiotics are administered in lower doses to mitigate toxicity [16]. SICs are particularly important in the context of biofilm-related infections because they can modulate bacterial gene expression, leading to the induction of stress responses [17], virulence [18], quorum sensing [18,19] and further biofilm formation [20,21].

eDNA is the most abundant polymer in the *P. aeruginosa* biofilm matrix and its production levels vary across strains. It plays a critical role by mediating cell-cell and cell-matrix interactions, thereby stabilising the biofilm architecture [22]. There is no universal mechanism for eDNA production across different species. However, in the majority of cases eDNA is derived from genomic DNA released into the extracellular milieu as a consequence of cell death, a process that promotes biofilm formation by supporting the growth and adhesion of the surviving community [22]. During the early stages of *P. aeruginosa* biofilm formation, eDNA is predominantly driven by explosive cell lysis, a programmed cell death mechanism mediated by the Lys endolysin, encoded within the R-pyocin and F-pyocin gene clusters [23]. Pyocins are bacteriocins produced by *P. aeruginosa*, which contribute to interbacterial competition and survival within polymicrobial environments, but their associated lytic components also facilitate eDNA release [24,23]. As the biofilm matures, additional regulatory systems come into play. Quorum sensing (QS) and the activity of the filamentous Pf4 phage both trigger lysis events in a subpopulation of cells, maintaining eDNA production during later biofilm stages [25,26]. Other mechanisms also cause cell lysis and subsequent eDNA release via flagella and type IV pili [26]. Furthermore, eDNA release in *P. aeruginosa* also occurs through oxidative stress caused by hydrogen peroxide (H_2_O_2_) generation mediated by pyocyanin production [27]. Pyocyanin is a redox-reactive phenazine molecule that is produced by 90–95% of *P. aeruginosa* strains and is present in high concentrations in CF lung infections [28]. These coordinated cell death mechanisms ensure continuous structural reinforcement and adaptation of the biofilm community.

Although a consensus has emerged on the role of many biofilm-associated traits, the large number of uncharacterized genes reported as differentially expressed under biofilm conditions highlights the substantial gaps that remain in our understanding of this complex bacterial lifestyle [29]. Among the upregulated loci in dormant, biofilm-dwelling *P. aeruginosa* cells is the small, uncharacterised gene *PA3049* [11], annotated as a homolog of the *Escherichia coli* ribosome modulator factor (*RMF*) [11]. RMF, a ribosomally associated protein, facilitates ribosome hibernation by associating with 100S ribosome dimers and modulates *E. coli* translation during the stationary phase [30]. However, while RMF is crucial for ribosome hibernation in *E. coli* [31], previous studies have demonstrated that, surprisingly, PA3049 does not fulfil this function in *P. aeruginosa* [32]. Despite its conservation in sequenced *P. aeruginosa* strains and presence in biofilm cells, the role of *PA3049* in *P. aeruginosa* biofilm formation is currently unknown [32].

To identify possible alternative functions for *PA3049*, we investigated its role in *P. aeruginosa* biofilm formation and responses to antibiotic challenge. Phylogenetic analysis showed that *PA3049*-like genes are widespread among γ-proteobacteria, with a substantial degree of sequence and structural divergence predicted between Pseudomonadales and Enterobacterales. We show that PA3049 plays an important role in shaping biofilm architecture and enabling established biofilms to withstand sub-inhibitory antimicrobial challenge. Proteomic and phenotypic analyses linked PA3049 activity to pyocyanin and R2-F2 pyocin production, both systems involved in biofilm development. PA3049 interacts directly with the uncharacterised Ser/Thr kinase PA0486 (SrkA), which also modulates pyocyanin levels, biofilm formation, and cell lysis, suggesting an *in vivo* regulatory connection.

Finally, the clinical relevance of PA3049 function was demonstrated using two clinically validated infection models: an established *ex vivo* pig lung (EVPL) model for *P. aeruginosa* biofilm infection of CF bronchioles [33], and the synthetic chronic wounds model (SCW) for *P. aeruginosa* diabetic foot infections [34].

Taken together, our findings indicate that PA3049 has diverged from the ribosome-associated proteins found in Enterobacteria and functions as a specialised, posttranslational regulator of biofilm formation and stress adaptation in *P. aeruginosa*. Given its key role in shaping the structure of *P. aeruginosa* biofilms, we renamed PA3049 as BatR (Biofilm architecture Regulator).

## Results

### *PA3049* homologs are widespread in γ-proteobacteria

**Fig 1:**
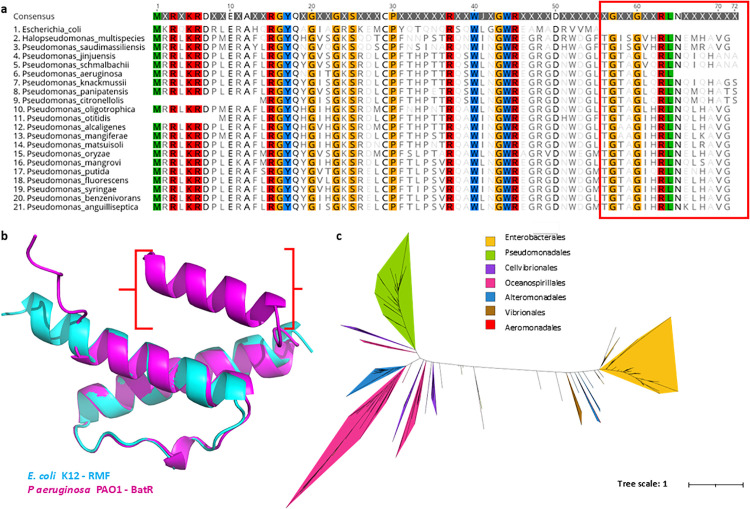
BatR is phylogenetically distinct from RMF. **a. Sequence alignment of BatR and RMF.** ClustalW alignment of RMF from *E. coli* str. K-12 substr. MG1655 and BatR from 20 different *Pseudomonas* spp. Different colours mark conserved residues in all 21 proteins. The 15-residue C-terminal extension on BatR sequences that is absent from RMF is shown in the red box. **b. AlphaFold 3 model of PAO1 BatR** (magenta), overlaid onto the structure of *E. coli* RMF (cyan). The additional alpha-helix at the C-terminus of the predicted BatR structure is indicated in red. **c. The phylogenetic relationship between BatR/RMF homologs.** The tree is based on 765 γ-proteobacteria homologous and was constructed using the maximum likelihood method, with 100 bootstrap replicates.

To uncover the role of PA3049 (BatR) in *P. aeruginosa*, we first analysed the distribution of *batR*-like genes in bacterial genomes, and compared its predicted structure to *E. coli* RMF, which shares ~49.09% sequence similarity with PA3049. Alignment of *batR* homologs from different *Pseudomonas* species with the *RMF* sequence from *E. coli* shows a 15-residue C-terminal extension encoded by *batR* that is absent from *RMF* (Fig 1a). AlphaFold3 [35] three-dimensional protein structure prediction of *P. aeruginosa* BatR highlighted a marked difference in the C-terminal fold compared to its *E. coli* homolog. Specifically, BatR contains a predicted 12 residue α-helix at the C terminus (red box), in addition to the two α helices connected by a 13-amino acid linker region that characterise *E. coli* RMF (Fig 1b).

A phylogenetic analysis of *batR* homologs revealed that these genes are confined to the γ-proteobacteria class (Fig 1c), with distinct evolutionary paths across diverse bacterial orders.

Notably, *batR* in Pseudomonadales forms a distinct cluster that diverges significantly from homologs in Enterobacterales, including *E. coli* and *Yersinia pestis*; and Vibrionales, including *Vibrio cholerae,* where RMF plays a role in ribosome hibernation [36]. Unlike other characterised RMF genes, *batR* deletion mutants do not exhibit impaired ribosomal integrity during starvation [32]. Thus, while *rmf* and *batR* are likely to share an ancestral root, their divergent structure and phylogeny (Fig 1) are consistent with an alternative functional role for BatR [32].

### *batR* protects *P. aeruginosa* biofilms from sub-inhibitory concentrations (SIC) of antibiotics *in vitro*

Given the heightened abundance of *batR* transcripts in dormant *P. aeruginosa* cells within biofilms [11], we assessed the importance of *batR* in biofilm-linked antimicrobial sensitivity *in vitro*. To do this, we generated a non-polar deletion mutant (Δ*batR*) in *P. aeruginosa* PAO1 (hereafter, PAO1) and examined its response to antimicrobial agents when grown under biofilm conditions. Assays were conducted using antibiotics targeting distinct metabolic processes, i.e., β-lactams (piperacillin, PIP) and quinolones (ciprofloxacin, CIP).

**Fig 2:**
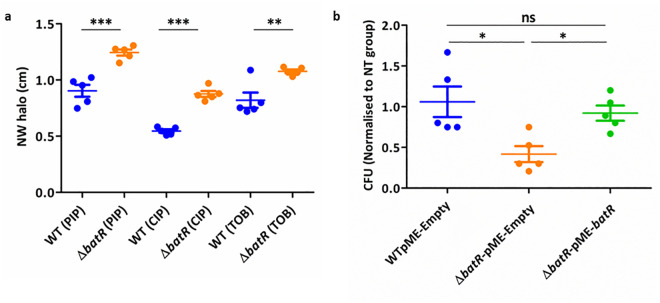
*batR* influences biofilm-associated antimicrobial sensitivity. **a. Antibiotic disk diffusion assay.** Results are shown as the normalized width of the antimicrobial halo (NW halo), calculated as described in [39]. Results were analysed by a one-way ANOVA and inhibition differed significantly between WT and ∆*batR* strains (F_5,24_ = 35.99, p < 0.0001). Tukey's multiple comparisons between WT and ∆*batR* strain under PIP, CIP and TOB treatments are indicated (p < 0.001 **, p < 0.0001 ***). **b. Glass Beads Biofilm survival**. Bacterial recovery (CFU/bead) from established biofilms grown on glass beads for 24h following treatment with SIC of PIP for 90 min. Strains used were WT PAO1 carrying the empty vector pME6032 (WT-pME-Empty), ∆*batR* carrying pME6032 (∆*batR*-pME-Empty), and ∆*batR* overexpressing *batR* (∆*batR*-pME-*batR*). Normalised values represent the estimated proportion of cells surviving PIP exposure, as described previously [38]. Results were analysed by a one-way ANOVA showing significant differences (F2,12 = 6.414; p < 0.05 *). Tukey's multiple comparisons between WT-pME6032 (PIP) and ∆*batR*-pME6032 (PIP), and between ∆*batR*-pME6032 (PIP) vs ∆*batR*-pME-*batR* (PIP) are indicated (p < 0.05 *).

The minimal inhibitory concentration (MIC) [37] of the tested antibiotics for cells grown in liquid culture was unaffected in ∆*batR*, (64 µg/mL for piperacillin and 0.5 µg/mL for ciprofloxacin). Interestingly however, Δ*batR* showed a small but significant increase in sensitivity to both PIP and CIP (12–17%), when grown on a solid surface (Fig 2a). We further confirmed the previously observed effect of the aminoglycoside tobramycin (TOB) ([11], referred to as *rmf*), with Δ*batR* again showing a significant increase in sensitivity on agar (Fig 2a). Next, we used a glass bead biofilm model [38], to assess the survival of cells within established biofilms challenged with PIP. This model revealed a substantial reduction in the survival of established Δ*batR* biofilms compared to WT PAO1, for samples exposed to a sub-inhibitory concentration (SIC) of PIP (Fig 2b). Furthermore, the Δ*batR* phenotype could be fully rescued by the heterologous expression of *batR* (Fig 2b). Growth curves confirmed that the presence of the plasmids pME-empty and pME-*batR* did not affect growth of the strains WT and Δ*batR* in shaking cultures (S1a Fig.).

Since the antibiotics tested in this study have different modes of action, our results suggest that BatR affects *P. aeruginosa* antimicrobial sensitivity through a general, rather than drug-specific mechanism. A simple explanation was a nonspecific change in membrane permeability. However, we ruled this out by measuring the intracellular concentration of resazurin, a fluorescent dye used to assess permeability and efflux (S2 Fig), which showed little difference between WT and ∆*batR* strains.

### BatR induces specific changes in the PAO1 proteome

To investigate the physiological changes associated with BatR in *P. aeruginosa* biofilms, we conducted a comparative proteomic analysis between the PAO1 WT and Δ*batR* strains. Strains grown on agar plates have been shown to function as organised and coordinated bacterial communities that share multiple characteristics with biofilms [10]. Therefore, whole-cell lysates were analysed by TMT proteomics following growth as a lawn on solid medium. On average, 4,581 individual proteins were detected per sample (S1 es), representing ~80% of the predicted total *P. aeruginosa* PAO1 proteome [40]. Surprisingly, the overall differences between WT and Δ*batR* proteomes were limited (S1 Data), suggesting that the impact of *batR* deletion is quite specific under the conditions tested.

Of the 21 proteins decreased in the Δ*batR* strain (Table 1), seven (PA0617-PA0633) comprise the R2/F2 pyocin gene locus [41]. In addition, the loss of *batR* led to reduced abundance of proteins involved in transport & metabolism, two heat shock proteins, and four proteins of unknown function. Conversely, the loss of *batR* increased the abundance of 14 proteins, including Rubredoxin-1, Type VI secretion system components and the Phenazine-1-carboxylate N-methyltransferase PhzM (Table 2).

**Table 1:** COG pathway analysis of proteins decreased in the Δ*batR* strain (TMT proteomics).

Locus	Gene	Product	COG
**General function prediction only**			
PA0617		Probable bacteriophage protein	General function prediction only
PA0618		Probable bacteriophage protein	General function prediction only
PA0619		Probable bacteriophage protein	General function prediction only
PA0620		Probable bacteriophage protein	General function prediction only
PA0623		Probable bacteriophage protein	General function prediction only
PA0626		Phage tail protein	General function prediction only
PA0633		Phage late control D family protein	General function prediction only
PA0803		VOC domain-containing protein	Function unknown
PA2853	*oprI*	Major outer membrane lipoprotein (Outer membrane lipoprotein I)	Function unknown
PA3796		DUF937 domain-containing protein	Function unknown
			
**Transport & Metabolism**			
PA3224		3-phosphoglycerate kinase	Metabolism
PA1625		AI-2E family transporter	
PA1906		HIT family protein	Nucleotide transport and metabolism; Carbohydrate transport and metabolism
PA2594		PBPb domain-containing protein	Inorganic ion transport and metabolism
PA2941		VWFA domain-containing protein	Coenzyme metabolism
PA3280	*oprO*	Porin O	Inorganic ion transport and metabolism
PA3113	*trpF*	N-(5’-phosphoribosyl) anthranilate isomerase (PRAI)	Amino acid transport and metabolism
**Translation, Posttranslational modification, protein turnover, chaperones**			
***PA3049***	***rmf/batR***	***Ribosome modulation factor/ Biofilm architecture regulator***	***Translation, ribosomal structure, and biogenesis***
PA5195	*yrfH*	Heat shock protein 15	Translation, ribosomal structure, and biogenesis
PA4762	*grpE*	Protein GrpE (HSP-70 cofactor)	Posttranslational modification, protein turnover, chaperones
**Cell envelope biogenesis, outer membrane -- Cell motility and secretion**			
PA1041		Probable outer membrane protein	Cell envelope biogenesis, outer membrane; Cell motility and secretion

**Table 2:** COG pathway analysis of proteins increased in the Δ*batR* strain (TMT proteomics).

Locus	Gene	Product	COG
**Energy production and conversion**			
PA5351	*rubA1*	Rubredoxin-1 (RdxS)	Energy production and conversion
**Lipid Metabolism**			
PA0286	*desA*	Delta-9 fatty acid desaturase	Lipid metabolism
PA3157	*wbpC*	Probable acetyltransferase	Lipid metabolism
**Type VI secretion system**			
PA1658	*hsiC2*	Type VI secretion system contractile sheath large subunit	Intracellular trafficking, secretion, and vesicular transport
PA2366	*hsiC3*	Uricase PuuD	Intracellular trafficking, secretion, and vesicular transport
PA2367	*hcp*	Type VI secretion system tube protein	Intracellular trafficking, secretion, and vesicular transport
PA0071	*tagR1*	FGE-sulfatase domain-containing protein	Hcp secretion island I (HSI-I) type VI secretion system
**Secondary metabolites biosynthesis, transport, and catabolism**			
PA4209	*phzM*	Phenazine-1-carboxylate N-methyltransferase	phenazine biosynthetic process
PA4143	*cyaB*	Probable toxin transporter	transmembrane transport
PA1436		Probable Resistance-Nodulation-Cell Division (RND) efflux transporter	Defence mechanisms; Inorganic ion transport and metabolism
PA2361		IcmF-related_N domain-containing protein	Intracellular trafficking, secretion, and vesicular transport
**Signal transduction mechanisms**			
PA4154	*ygiM*	SH3b domain-containing protein	Signal transduction mechanisms
**Unknown function**			
PA4297	*tadG*	TadG	Function unknown
PA2030		Transmembrane protein	Function unknown

In *P. aeruginosa* PAO1, the R2/F2 pyocin cluster encodes the structural components for R2 and F2 pyocins, a shared regulatory region and a lytic cassette made up of four proteins (S3a Fig). Among these, the endolysin Lys (PA0629) and the holin (PA0614) are essential for explosive cell lysis, a process in which a subset of cells transition from rod-shaped to round then lyse, releasing intracellular contents including genomic DNA, critical for early biofilm development [23,42]. TMT proteomics revealed increased levels of several R2-type pyocin proteins (PA0617, PA0618, PA0619, PA0620, PA0623 and PA0626) and an F2-type pyocin (PA0633) in the WT strain, suggesting BatR-dependent regulation of the pyocin cluster (S3a Fig).

To validate these assays, the promoter activity of *PA0614*, the first gene of the R2/F2 pyocin gene cluster, was measured (S3b Fig). We use the pM0614-G plasmid, in which the *PA0614* promoter is fused to an *egfp* reporter, and a promoterless pMEXGFP plasmid as a negative control [43]. In the Δ*batR* mutant, *PA0614* promoter activity was significantly decreased compared to the WT, supporting our proteomic results that BatR acts as a positive regulator of pyocin production (whether directly or indirectly). Then, to directly analyse the effect of BatR on explosive lysis, we performed phase-contrast time-lapse microscopy using a microfluidic device [44]. We quantified lysis events based on the transition of rod-shaped cells into round morphotypes followed by lysis over a 3h period. Under these conditions, no significant differences in lysis frequencies were observed between WT and Δ*batR* strains (S3b, c Fig). Nonetheless, we cannot rule out a BatR-dependent effect on explosive lysis under different growth conditions. BatR appears to function primarily in biofilms, and may not be present or active under the low-density, flow-based conditions of the microfluidic setup.

### BatR interacts with a putative stress response kinase

**Fig 3:**
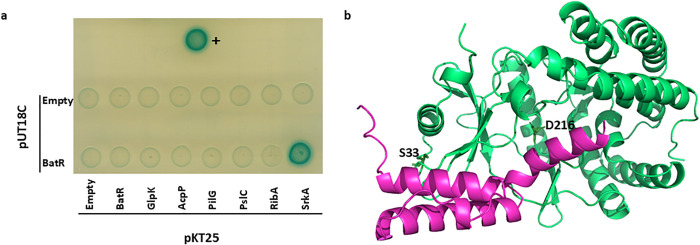
BatR interacts with a predicted kinase protein. **a.** Representative image of qualitative β-galactosidase assays on agar plates. pKT25 and pUT18C fusions are shown in rows and columns, with the indicated protein/empty vector present in each case. Positive control (+): pKT25-zip and pUT18C-zip encoding the two adenylate cyclase fragments, T25 and T18 [46]. **b.** Predicted interface between the Alphafold3 models of BatR (magenta) and SrkA (green). The conserved residues Ser33 (S33), predicted ATP-binding site, and Asp216 (D216), predicted Mg2 + -binding site, in SrkA are shown.

To understand the molecular basis of BatR function, we next investigated its interactions with other PAO1 proteins by performing a co-immunoprecipitation (Co-IP) analysis (S2 Data). BatR-3xFlag co-immunoprecipitated alongside several cytoplasmic proteins, suggesting potential direct regulatory influences (Table 3). Independent validation of strongly co-precipitating proteins was conducted using Bacterial Two-Hybrid (B2H) analysis. This confirmed specific interaction between BatR and the predicted kinase PA0486, annotated as Stress response kinase A (SrkA) [45](Fig 3a).

**Table 3:** BatR interacting proteins (Co-IP assay).

Accession	Gene	log fold change	apv	Product
**Q9HZF9**	batR	6.643856	2.28E-15	**BatR (bait protein)**
Q51390	glpK2	**6.643856**	**2.28E-15**	Glycerol kinase 2
O54439	acpP1	6.643856	2.28E-15	Acyl carrier protein 1
Q9I1N6	pslC	6.150153	1.05E-10	PslC
P46384	pilG	5.064366	2.47E-07	Protein PilG
Q9HWX6	*nusB*	4.297705	2.28E-04	Transcription antitermination protein NusB
Q9HWY1	ribA	3.912171	6.89E-04	GTP cyclohydrolase-2
Q9HZG2	*PA3046*	3.722466	0.00102	Uncharacterized protein
Q9I4Q5	*PA1069*	3.65581	5.92E-04	Uncharacterized protein
Q9HXI8	*iscS*	3.505764	0.00102	Cysteine desulfurase IscS
Q9I0X9	*PA2503*	3.199751	0.006032	Uncharacterized protein
P42810	*PA4667*	3.114367	0.009501	TPR repeat-containing protein PA4667
Q9HZV0	*PA2897*	3.068155	0.006658	Probable transcriptional regulator
Q9HXI7	*iscR*	3.02556	0.014421	IscR
Q9I5R6	*coq7*	3.015605	0.007976	3-demethoxyubiquinol 3-hydroxylase
Q9HVX2	*PA4445*	3.013998	0.01486	GTP cyclohydrolase 1 type 2 homolog
Q9HYZ5	*minE*	2.710614	0.04354	Cell division topological specificity factor
Q9I513	*purM*	2.709291	0.021917	Phosphoribosylformylglycinamidine cyclo-ligase
O52761	*rplQ*	2.704429	0.021917	50S ribosomal protein L17
Q9HV50	*glmM*	2.571677	0.034564	Phosphoglucosamine mutase
Q9I0H4	*hmp*	2.564622	0.04354	Flavohemoprotein1
Q9HWE0	*rplV*	2.561937	0.034689	50S ribosomal protein L22
Q9I6G3	*PA0328*	2.541515	0.046616	Autotransporter domain-containing protein
Q9HUG9	*hldE*	2.540275	0.04354	Bifunctional protein HldE
**Q9I632**	srkA	**2.486714**	**0.04354**	**Stress response kinase A**
Q9HWF1	*rplR*	2.447844	0.04354	50S ribosomal protein L18

To gain additional insight into the potential interaction between BatR and SrkA, we used AlphaFold3 [35] to model the potential interactions of BatR with the target protein (Fig 3b). Whilst the Alphafold3 interaction prediction for BatR had low confidence (0.270), the prediction did indicate a potential interaction with the active site of SrkA, including the residue D216, predicted to be the Mg^2+^ binding site.

### SrkA influences biofilm formation, cell death, and pyocyanin production in *P. aeruginosa*

SrkA (PA0486) is annotated as a eukaryotic-like serine-threonine protein kinase. Its homolog in *E. coli* is implicated in protecting cells from stress-induced programmed cell death [45,47]. Since SrkA remains uncharacterized in *P. aeruginosa*, we overexpressed *srkA* in both the WT and Δ*batR* PAO1 strains to assess its functional role. To test whether the observed phenotypes are dependent on SrkA’s kinase activity, we also overexpressed a catalytically inactive (“null”) version of *srkA*, in which the conserved Ser33 and Asp216 residues were substituted with alanine. Alphafold3 analysis of the predicted interaction between BatR and SrkA suggests that BatR binding may interfere with Ser33, which is predicted to be the ATP-binding residue (Fig 3b).

**Fig 4:**
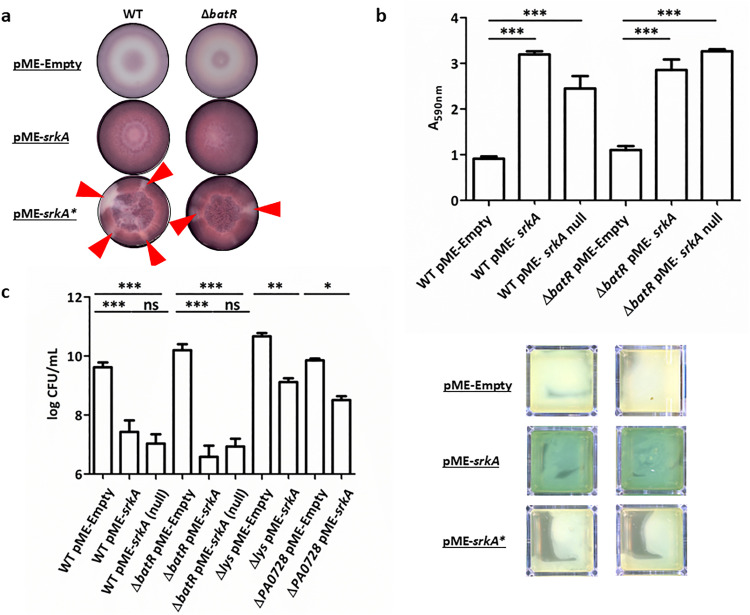
Phenotypic effects of *srkA* overexpression in PAO1 WT and ∆*batR* strains. **a. Congo-red binding.** Representative images of WT-pME-Empty, WT-pME-*srkA*, WT-pME-*srkA* null, ∆*batR*-pME-Empty; ∆*batR*-pME-*srkA* and ∆*batR*-pME-*srkA* null biofilms grown using the colony biofilm morphology assay. Only plates supplemented with IPTG are shown (7 days). Escape mutants are indicated with red arrows. **b. Biofilm formation.** Strains were grown statically in LB medium for 24h at 37°C. Biofilm biomass was quantified by Crystal Violet staining and measured spectrophotometrically at 590 nm (A_590nm_). Values represent the mean of five biological replicates with two technical replicates each; error bars indicate SD. **c. Cell viability.** Cells were scraped from LB agar plates, resuspended in PBS, and enumerated via serial dilution and plating. **d. Pyocyanin production.** Top view images of cell lawns showing pyocyanin production (blue-green colouration). Results were analysed by a one-way ANOVA showing significance between strains in **b** (F_5,54_ = 45.17, p < 0.0001) and **c** (F_7,32_ = 28.01, p < 0.0001). Tukey's multiple comparisons between strains are indicated (p < 0.05 *, p < 0.001 **, p < 0.0001 ***).

Overexpression of *srkA* led to an increase in biofilm formation in both solid (Congo red binding) and liquid (Crystal Violet staining) media (Fig 4a, b), as well as an increase in pyocyanin production (Fig 4d). This increase in biofilm was independent of the SrkA kinase activity, with the overexpression of the SrkA null mutant showing identical phenotypes to the WT version of SrkA. Overexpression of the kinase-inactive variant also produced a distinctive, highly aggregative colony phenotype on solid media and the emergence of multiple escape mutants, manifesting as regions of reduced dye binding and WT PAO1 morphology (Fig 4a).

Curiously, the overexpression of *srkA* led to a kinase-independent significant increase in cell death, with even a small induction of SrkA leading to a 10^3^-fold reduction in cell viability. Growth curves confirmed this *srkA*-killing effect in shaking cultures, with impaired growth in strains overexpressing *srkA* and more pronounced effect in strains overexpressing the null version of *srkA* (S1 b Fig.).

Given the link between BatR and the R2/F2 pyocin cluster suggested by the proteomic data, we hypothesised that the *srkA*-mediated killing effect occurs through a similar mechanism. To test this, we overexpressed *srkA* in a mutant deficient in production of the endolysin Lys (PA0629) [23]. While the impact of *srkA* expression was markedly reduced from that seen in WT PAO1, the ∆*lys* pME-*srkA* mutant still exhibited an approximately 10^2^-fold reduction in viable cell counts compared to the empty vector control (Fig 4c). This suggests that *srkA* induced cell death is partially endolysin-dependent but also implicates an additional lethal pathway that operates independently of the R2/F2 pyocin lysis system.

We next generated a non-polar deletion mutant (Δ*srkA*) and the double mutant (Δ*batR* Δ*srkA*) in *P. aeruginosa* PAO1 and examined their phenotypes. Interestingly, despite SrkA’s dramatic effect when overexpressed, Δ*srkA* showed no phenotypic differences from WT at baseline under the conditions tested. However, Δ*batR* Δ*srkA* displayed several strong phenotypes: reduced biofilm formation, increased cell death and increased pyocyanin production (S4 Fig). Growth curves confirmed that the deletion of *batR* and *srkA* did not result in growth impairment in shaking cultures (S4 c Fig.). The fact that these traits emerge only in the absence of both genes is consistent with an additional, unidentified regulator whose activity is suppressed by BatR and SrkA.

### SrkA activates multiple cell death mechanisms in *P. aeruginosa*

**Fig 5:**
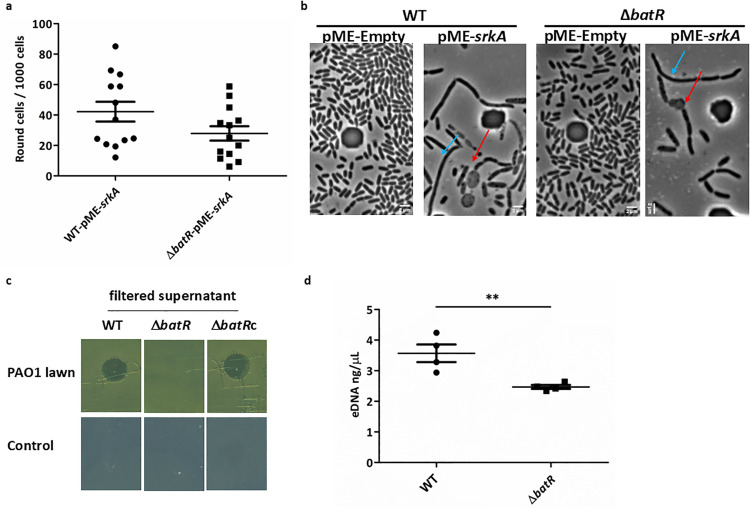
a. *srkA* overexpression increases explosive cell lysis in *P. aeruginosa.* Cells were classified as rod-shaped or round over an 11-hour period. The frequency of round cells was calculated as the number of round cells divided by the total number of cells counted at the final time point. No significant differences were observed between strain backgrounds under the conditions tested. **b.** *srkA* **overexpression alters cell morphology in *P. aeruginosa***. Phase contrast images from an 11h microfluidics assay of WT pME-Empty, pME-*srkA* ∆*batR*, pME-Empty and pME-*srkA* strains. Cells were incubated for 1h in LB, followed by 9h in LB + 0.1mM IPTG, and a final 1 h in LB. Each experiment was performed independently at least three times. Two primary phenotypes were observed upon *srkA* overexpression: increased frequency of explosive cell lysis (red arrows) and pronounced cell elongation (blue arrows). Time point: 11 h; scale bar: 2 μm. **c. Bacteriophage activity using cell-free supernatants from WT PAO1 and ∆*batR* strains**. **Top:** 20 µL drops from the filtered supernatants of WT, ∆*batR,* and ∆*batR*c (∆*batR*::*batR*) PAO1 strains were spotted onto the indicator strain, WT PAO1. Zones of clearing on the indicator lawn indicate cell lysis. **Bottom**: a control LB agar plate was spotted with 20 µL of each supernatant to ensure they were free of bacterial growth. Plates were incubated at 37°C overnight. **d. Extracellular DNA quantification**. Double stranded extracellular DNA (eDNA) concentration in the filtered supernatants was quantified on a Qubit Fluorometer using the high sensitivity dsDNA assay kit. The bars denote means of five biological replicates and a T-test shows significant differences between WT and ∆*batR* (t = 3.735 df = 6, p = 0.0097)*.*

To visualise the SrkA-mediated killing effect, we repeated the phase-contrast time-lapse microscopy using a microfluidic device. Lysis events increased from 2-3 per 1,000 cells in the control strains (WT and ∆*batR*) (S3 c Fig) to 30–40 per 1,000 cells upon *srkA* overexpression (Fig 5a). Overexpressing cells appeared highly compromised, with frequent round cells and evidence of explosive lysis (Fig 5b). Additionally, *srkA* overexpression induced distinct morphological changes, including the emergence of highly elongated filamentous cells (Fig 5b).

To gain further insight into the SrkA mechanism of action, we re-isolated six escape mutants -three from WT PAO1-pME-*srkA* null and three from ∆*batR* PAO1-pME-*srkA* null (Fig 4a) and subjected them to whole-genome sequencing. Comparative analysis of escape mutant genomes revealed that all three mutants derived from the ∆*batR* background had deleted the *srkA* null gene from the overexpression plasmid. In the WT background, one of the three mutants harboured a plasmid-*srkA* null deletion, while a second contained a single-nucleotide polymorphism (SNP) that introduced a premature stop codon at position 147 of the *srkA* null open reading frame, resulting in a truncated and likely nonfunctional protein. The third WT-derived escape mutant sequence harboured multiple unique SNPs in the chromosome, but not in the plasmid, suggesting that bypassing *srkA* null toxicity (without inactivating the gene itself) involves multiple genetic changes. Interestingly, many of these SNPs were in genes encoding oxidoreductases, biofilm-regulators (*gacA, bfmS*) [48], and DNA repair proteins.

We detected a substantial number of SNPs within the *PA0717-PA0727* gene cluster, which encodes components of a Pf1-like filamentous bacteriophage (Pf4) in all six strains. This mutational enrichment coincided with increased read depth across the region, consistent with active replication of the prophage (S5 Fig). This observation is notable, as phage Pf4 has previously been implicated in promoting cell death and eDNA release in mature PAO1 and PA14 biofilms [25,49,50]. Pf4 is unusual among bacteriophages because it lacks a canonical endolysin and is capable of infecting cells within the clonal population from which it originates [49]. Furthermore, the highly elongated filamentous cells observed during *srkA* overexpression (Fig 5b), resemble phenotypes associated with Pf4 activity during biofilm development [51]. Together, these findings suggest that Pf4 may represent the additional SrkA-triggered lethal pathway that operates independently of the classical R2/F2 pyocin lysis system. To test this hypothesis, we overexpressed *srkA* in PAO1 ∆*PA0728,* which lacks the Pf4 integrase [52]. Similar to the ∆*lys* mutant, the effect of *srkA* overexpression on cell viability was attenuated in ∆*PA0728* strain compared to WT PAO1 (Fig 4c). These results indicate that *srkA*-induced cell death is mediated through at least two distinct pathways, with contributions from both endolysin-dependent lysis and a Pf4-associated mechanism.

We therefore asked whether BatR also contributes to Pf4 production or activity. To test this, WT and Δ*batR* strains were grown under conditions used for the proteomic assays and filtered culture supernatants were spotted onto lawns of PAO1 WT cells. Interestingly, only WT supernatants produced plaques, whereas Δ*batR* supernatants did not (Fig 5c). This phenotype was fully rescued by heterologous expression of *batR* (Δ*batR*c), implicating BatR in phage-mediated cell death and eDNA release (Fig 5c). No differences in growth between these strains were observed in shaking cultures (S1c Fig.). Proteomic analysis of these supernatants identified the Pf4 coat protein A among the most enriched proteins in WT samples (Table 4, S3 Data). Consistent with this, quantification of eDNA revealed significantly higher levels in WT supernatants compared to those from the Δ*batR* strain (Fig 5d).

**Table 4:** Proteins increased in WT supernatants (Label free proteomics).

Locus	Product
PA4831	Probable transcriptional regulator
PA0724	Coat protein A of bacteriophage Pf1
PA3017	Universal stress protein
PA3901	Fe(III) dicitrate transport protein FecA
PA2795	tRNA-dihydrouridine synthase A
PA5082	Probable binding protein component of ABC transporter
PA0599	DUF3530 family protein
PA1410	Probable periplasmic spermidine /putrescine-binding protein
PA5227	Cell division protein ZapA (Z ring-associated protein ZapA)
PA2800	VacJ family lipoprotein
PA3762	NGG1p interacting factor NIF3
PA0792	2-methylcitrate dehydratase
PA4278	SPOR domain-containing protein
PA4447	Histidinol-phosphate aminotransferase 1 (Imidazole acetol-phosphate transaminase 1)
PA0228	Beta-ketoadipyl-CoA thiolase (3-oxoadipyl-CoA thiolase)
PA1339	Amino acid ABC transporter ATP binding protein
PA0862	TenA family transcriptional regulator
PA1674	GTP cyclohydrolase 1 2
PA3022	MOSC domain-containing protein
PA0729	Type II toxin-antitoxin system RelE/ParE family toxin
PA4741	30S ribosomal protein S15
PA2275	Probable alcohol dehydrogenase (Zn-dependent)
PA0052	Cupin domain-containing protein
PA3240	Putative quercetin 2,3-dioxygenase PA3240 (Putative quercetinase) (Pirin-like protein PA3240)
PA0328	Autotransporter domain-containing protein
PA3655	Elongation factor Ts (EF-Ts)
PA4670	Ribose-phosphate pyrophosphokinase (RPPK)
PA4135	Probable transcriptional regulator
PA0904	Aspartokinase
PA1714	Transcriptional antiactivator ExsD
PA0077	Type VI secretion system component TssM1
PA2082	Probable transcriptional regulator
PA3246	Pseudouridine synthase RluA
PA2756	3-isopropylmalate dehydratase large subunit
PA0402	Aspartate carbamoyltransferase
PA1204	NAD(P)H-dependent FMN reductase
PA0849	Thioredoxin reductase
PA4653	Spore coat protein U
PA4217	5-methylphenazine-1-carboxylate 1-monooxygenase
PA4874	Phosphate starvation-inducible protein PsiF
PA0766	Periplasmic serine endoprotease DegP-like
PA4481	Cell shape-determining protein MreB
PA4650	SCPU domain-containing protein
PA1252	Delta(1)-pyrroline-2-carboxylate reductase
PA0660	NMO domain-containing protein
PA0359	Multidrug transporter
PA3997	Octanoyltransferase (Lipoate-protein ligase B)
PA2023	UTP--glucose-1-phosphate uridylyltransferase
PA5520	Tetratricopeptide repeat protein
PA5263	Argininosuccinate lyase
PA0623	Probable bacteriophage protein
PA4847	Biotin carboxyl carrier protein of acetyl-CoA carboxylase (BCCP)
PA1272	Corrinoid adenosyltransferase
PA5257	HemY_N domain-containing protein
PA5558	ATP synthase subunit b
PA0633	Phage tail protein
PA3416	Probable pyruvate dehydrogenase E1 component, beta chain
PA3813	Iron-sulfur cluster assembly scaffold protein IscU
PA1585	oxoglutarate dehydrogenase (succinyl-transferring)
PA3724	Elastase
PA5015	Pyruvate dehydrogenase E1 component (PDH E1 component) (EC 1.2.4.1)

Next, we used RT-qPCR to examine the influence of *batR* and *srkA* on the regulation of the R2/F2 pyocin operon and the Pf4 bacteriophage at a transcriptional level. RT-qPCR analysis of *PA0614* (*lys*) and one of the core genes of Pf4, *repG4* (*PA0717*), transcription showed very low expression levels in the WT and the single mutants but a strong upregulation in the double mutant ∆*batR* ∆*srkA*, consistent with phenotypes seen for this mutant (S4 e, f Fig., respectively).

### BatR affects the ability of *P. aeruginosa* biofilms to withstand antibiotic challenge in clinically validated infection models

Given BatR’s role in biofilm-mediated antibiotic sensitivity under laboratory conditions, we next determined whether this effect extends to clinically relevant infection models. First, we used an *in vitro* synthetic chronic wounds (SCW) model, which mimics the environment of diabetic foot infections [34], to assess biofilm formation following exposure to increasing concentrations of CIP (0–64 µg/mL).

**Fig 6:**
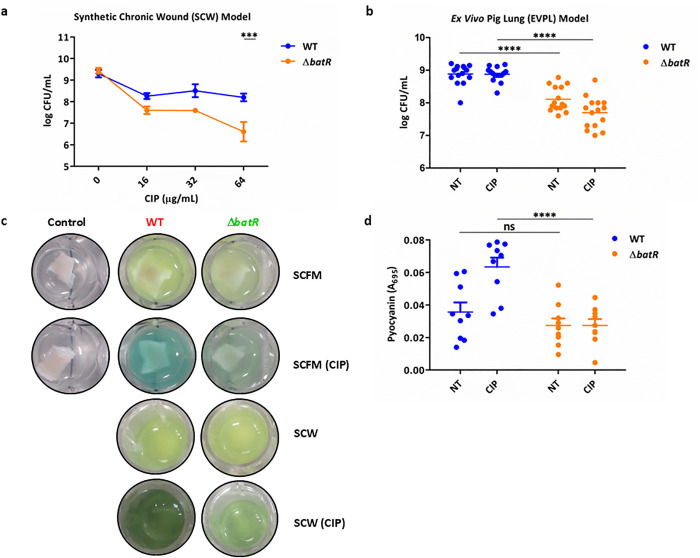
BatR contributes to antibiotic resistance and pyocyanin production in clinically validated models. **a. Synthetic Chronic Wounds (SCW) Model**. Viability of *P. aeruginosa* PAO1 and the Δ*batR* strain living in established biofilms (24 **h)**, treated with different concentrations of CIP in the cell suspension on top of the matrices. Dots represent the mean from three biological replicates and error bars indicate the standard deviation. Two-way ANOVA showed significant differences for WT and Δ*batR* under 64 µg/mL CIP treatment (p < 0.001 **). **b. *Ex Vivo* Pig Lung (EVPL) Model**. Growth of *P. aeruginosa* PAO1 strain and Δ*batR*, on 15 pieces of EVPL bronchiole (three replicate pieces of tissue from five independent lungs infected per strain) plus Synthetic Cystic Fibrosis Medium (SCFM), with and without 16 µg/mL CIP treatment [16]. Colony forming units (CFU) were retrieved from biofilms after 2 d growth at 37°C. Bars denote mean for each genotype across all five lungs, and asterisks denote a significant difference under that condition. Two-way ANOVA analysis showed significant differences for WT vs ∆*batR*; and WT + CIP vs ∆*batR* + CIP (p < 0.0001 ***). **c. Biofilm pigmentation.** The characteristic blue-green colouration of *P. aeruginosa* results from a mixture of the exoproducts pyoverdine and pyocyanin. Notably, pigmentation intensity was higher in the WT strain when grown on bronchiolar tissue sections in SCFM and as biofilm aggregates in a collagen gel matrix in the SCW model. **d. Quantification of pyocyanin by *P. aeruginosa* WT and ∆*batR* in the EVPL model.** Differences in pyocyanin production (A_695_) between WT and Δ*batR* under CIP treatment were significant (p < 0.0001***).

In the absence of antibiotic, no differences in biofilm formation were observed between the WT and ∆*batR* strains. However, upon CIP treatment, PAO1 ∆*batR* biofilms showed significantly reduced viavility compared to WT biofilms (Fig 6a), with a nearly 40-fold reduction in CFU recovery in the ∆*batR* mutant at higher CIP concentrations.

To expand these findings to a more complex system, we next used an *ex vivo* pig lung (EVPL) model that recapitulates *P. aeruginosa* biofilm infections in CF bronchioles [33]. This model comprises bronchiolar lung tissue and Synthetic Cystic Fibrosis Medium (SCFM) to replicate the luminal mucus environment [53]. Biofilms were challenged with CIP at a SIC (16 µg/mL, 1/8^th^ of the MIC in the EVPL model, S6 Fig) and CFUs were enumerated at 2 days postinfection (dpi).

CFU counts in WT biofilms were unaffected by CIP treatment. In contrast, ∆*batR* biofilm formation was reduced even in the absence of CIP, and further decreased upon CIP treatment, resulting in 2.5-fold reduction in bacterial load under antibiotic stress. Our results confirm that BatR contributes to CIP resistance and supports biofilm establishment within the EVPL model (Fig 6b).

Alongside these effects on biofilm formation, increased blue pigmentation, indicative of pyocyanin production, was evident in WT biofilms compared to Δ*batR* following CIP treatment (Fig 6c, d). A similar effect was also observed in WT biofilms relative to ∆*batR* in the synthetic chronic wound model under antibiotic stress (Fig 6c).

### BatR contributes to biofilm architecture

Next, given BatR’s role in promoting eDNA release under laboratory conditions, we examined the biofilm architecture in infected lung tissue using the EVPL model, with and without SIC of the antibiotic CIP. Infected lung pieces were collected in replica sets and fixed at 2- and 7-dpi, then paraffin-embedded. Sections were stained with H & E (Haematoxylin & Eosin) to visualise total biofilm mass and tissue structure. As expected, distinct biofilm structural differences were observed at both time points (S7 and 7a, b, Figs respectively) between ∆*batR* and WT strains.

At 2 dpi, the WT strain exhibited significantly higher staining intensity, indicative of denser and more extensive biofilm formation, both in the presence and absence of CIP (S7 Fig). This observation aligns with the higher viability counts observed for the WT strain (Fig 6b), suggesting a larger population of viable cells contributing to the biofilm mass.

**Fig 7:**
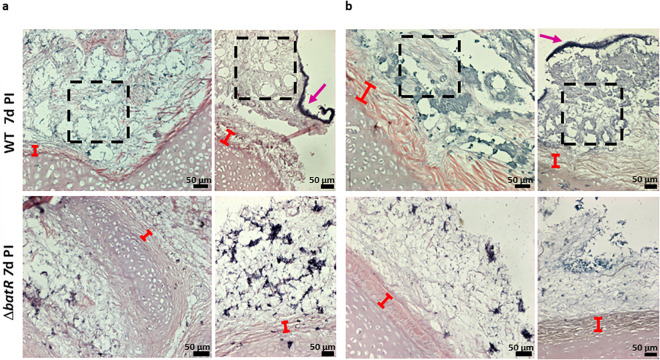
BatR contributes to biofilm architecture. **Haematoxylin and eosin (H & E) stained sections of EVPL bronchiolar tissue at 7 dpi.** EVPL was infected with *P. aeruginosa* PAO1 WT and ∆*batR*, with uninfected tissue as a negative control. The x20 magnification images from the sections are shown here for **a.** non-treated tissues (SCFM) and **b.** treated with CIP (SCFM + CIP). The cartilage and tissue surface (red bar) stain pink and the bacterial biofilm stain purple, including the bacterial cells and biofilm matrix. The typical sponge-like structure is shown by the dashed boxes on the WT images, and the purple arrow shows the thick layer of matrix covering the biofilm in WT. Representative images of phenotypes at 7 dpi are shown here, but the same results were observed for all biological replicates analysed.

By 7 dpi, the EVPL biofilms show a characteristic “sponge-like” appearance consisting of extracellular matrix punctuated by gaps, resembling CF biofilms observed *in vivo* [54,55,56]. Interestingly, qualitative differences in sponge-like architecture were apparent between the ∆*batR* and WT strains, as shown in Fig 7a, b. The WT biofilms showed a thicker matrix and more pronounced structural features, both in untreated samples and those exposed to SIC of CIP. These features were not observed in the ∆*batR* biofilms, suggesting that *batR* influence on biofilm architecture may explain the reduced structural resilience we observe in ∆*batR*, particularly under antibiotic stress (Fig 6b).

## Discussion

In this study we characterise the *P. aeruginosa* proteins BatR and SrkA and determine their contributions to biofilm formation, programmed cell death, pyocyanin production and antimicrobial sensitivity, highlighting in turn their potential clinical significance. *batR* homologs exhibit a high degree of conservation within diverse bacteria, particularly in γ-proteobacterial species [36]. The identification of a well-supported clade of Pseudomonadales carrying *batR* homologs that is both genetically and structurally distinct from the characterised Enterobacterales/Vibrionales *rmf* clade suggests a divergent evolutionary path for these genes and supports a distinct cellular function for BatR. The distinct physiological roles of BatR and Rmf showcase how homologous proteins can diverge significantly in function and structure despite sharing elements of sequence conservation, highlighting the importance of experimental validation when inferring protein function across species.

Our findings suggest that *P. aeruginosa* BatR has diverged from its common ancestor with *E. coli* Rmf to regulate phenotypes associated with biofilm formation and stress adaptation during chronic infection. Supporting this, *batR* deletion mutants exhibited a marked difference in biofilm development and an increased sensitivity to multiple antibiotics, including β-lactams and fluoroquinolones, under sessile growth conditions, such as those found in CF and chronic wound infections. Previous transcriptomic analysis of *P. aeruginosa* strain PA14 in EVPL model revealed significant differential expression of *batR* at 7- compared with 1-dpi [53]. In addition, *batR* is differentially increased in burn wound infections [57], underscoring the versatility of *batR* in mediating *P. aeruginosa* pathogenesis across various infection settings.

eDNA is a critical component of the *P. aeruginosa* biofilm matrix, playing a key role in biofilm development [22]. In this study, ∆*batR* formed biofilms in our EVPL model with distinct structural deficiencies compared to those produced by WT PAO1. We speculate that differences observed in biofilm development may be driven in part by differential eDNA release. Our proteomic data support this hypothesis, showing that one-third of the proteins increased in biofilms of the WT strain compared to the Δ*batR* strain are part of the R2-pyocin locus known to induce self-lysis [23].

BatR appears to function predominantly in biofilms. Little or no impact of *batR* deletion was observed for PAO1 growing in liquid culture or microfluidic chambers, and only relatively modest effects were seen on plates, where a subpopulation of cells is in a state similar to biofilm [58]. In contrast, during growth as a biofilm on abiotic surfaces or in clinical models, differences in biofilm development and architecture between WT and ∆*batR* seem to be critical for withstanding subinhibitory antibiotic concentrations.

In addition to its contribution to biofilm development, BatR function appears to be consistently associated with pyocyanin production. Pyocyanin is a critical virulence factor in chronic lung infections in CF patients, impacting gene expression, colony size and biofilm thickness [59]. This molecule interacts with O_2_ to form reactive oxygen species, such as hydrogen peroxide (H_2_O_2_), leading to redox imbalance and host cell injury [60]. Pyocyanin dependent H_2_O_2_ production also facilitates cell death that contributes to biofilm formation via the release of eDNA [61]. In addition, pyocyanin intercalates with eDNA, altering cell surface properties such as hydrophobicity and attractive surface energies, which promotes cell aggregation [62]. Within the oxygen-limited environment of *P. aeruginosa* biofilms, pyocyanin is crucial for metabolic continuity and significantly impacts the biofilm's response to antibiotic treatments [63,64,65].

Given the hydrophilic nature of BatR [66] and the absence of conserved regulatory domains, we hypothesised that BatR may function by direct interaction with other proteins. This led us to identify an interaction between BatR and PA0486 (SrkA). In *E. coli*, the SrkA kinase has been implicated in stress-induced programmed cell death [45], however its function in *P. aeruginosa* was unknown. We show that in addition to cell death responses mediated via induction of R2/F2 pyocin and bacteriophage Pf4, *srkA* overexpression positively regulates both biofilm formation and pyocyanin production in *P. aeruginosa*. Curiously SrkA appears to function via two discreet regulatory mechanisms: pyocyanin stimulation requires an active SrkA kinase, whereas the SrkA cell death and biofilm phenotypes were kinase independent.

Programmed cell death is a tightly regulated process typically associated with multicellular organisms, where it promotes organismal fitness by eliminating damaged or unnecessary cells [67,68]. Pyocin and phage-mediated cell lysis fulfil similar roles in bacteria, and can be viewed as a form of bacterial programmed cell death [23]. In this context, the biofilm acts as a multicellular community, where the release of public goods such as eDNA benefits the population. Our data suggest that BatR and SrkA modulate three key phenotypes in *P. aeruginosa* PAO1 biofilms: the production of biofilm components, enabling surface attachment and matrix formation; programmed cell death leading to eDNA release; and pyocyanin production, supporting redox chemistry [69]. The coordinated deployment of these traits enhances biofilm formation and contributes to population-level resilience.

As a small protein lacking enzymatic or DNA-binding domains, BatR likely acts as an allosteric partner for other regulators, influencing the activity of SrkA and potentially other proteins under specific conditions. Our findings support a model in which BatR interacts with SrkA to modulate its activity, and imply additional, unidentified regulatory partners for BatR and SrkA. If *batR* functioned exclusively upstream of *srkA* then we would expect the ∆*srkA* phenotype to be epistatic over *batR*. However, *srkA* deletion phenotypes are only observed in a ∆*batR* background, where ∆*srkA* strongly exacerbates the *batR* phenotype. This suggests the existence of another unidentified regulator, or regulators, whose activity is suppressed by both BatR and SrkA. When both *batR* and *srkA* are missing, this additional regulator may trigger the phenotypes we observe in S3 Fig. Identifying the remaining members of the BatR regulon, alongside the downstream targets of SrkA are high priorities for future research.

*P. aeruginosa* biofilms are a hallmark of chronic infection and mortality in CF patients and are associated with poor clinical outcomes. Mechanisms that regulate susceptibility to cell death within biofilms, such as those mediated by BatR and SrkA, represent attractive potential targets for therapeutic interventions aimed at disrupting biofilm integrity and improving treatment efficacy.

## Materials and methods

### Bioinformatic analysis

A phylogenetic tree of RMF proteins was constructed using 765 publicly available protein sequences from the NCBI database. The dataset was curated based on a criterion of 50% sequence identity and 40% query cover to ensure the representation of diverse homologs while maintaining a reasonable level of similarity. The multiple sequence alignment was performed using Clustal Omega (v1.2.4), generating an alignment matrix with 137 columns and 135 distinct patterns. Phylogenetic signal analysis revealed 82 parsimony-informative sites, 33 singleton sites, and 22 constant sites. Manual curation was conducted to remove repetitive sequences and false hits. The phylogeny estimation was done using IQ-TREE, (multicore v1.6.12), employing the maximum likelihood (ML) criterion. The tree visualization was done using iTOL (v6.8.1), representation chosen was an unrooted tree with branch lengths proportional to the inferred evolutionary distances between sequences. The clade colours were assigned at order level of the taxonomy.

### Bacterial strains and growth media

Bacterial strains and plasmids used in this study are listed in Table 5. Unless otherwise stated, *P. aeruginosa* PAO1 and *E. coli* DH5α strains were routinely cultured in LB (lysogeny broth) [70] at 37°C solidified with 1.5% w/v agar where appropriate. For growth curves, cells were grown at a starting OD_600_ of 0.01 in a 96-well plate. Measurements were taken every 60 min for up to 48 h on a FLUOstar nano plate reader (BMG) with the plate being incubated at 37 °C under planktonic conditions.

**Table 5:** Strains and plasmids used in this study.

Strain	Description	Reference
*P. aeruginosa*		
WT PAO1	Wild-type *Pseudomonas aeruginosa* PAO1	[71]
Δ*batR* PAO1	Non-polar deletion of *PA3049* (*batR*)	This study
Δ*lys* PAO1	PA0629 deletion mutant of PAO1	[23]
Δ*batR*c PAO1	Δ*batR* PAO1 containing *PA3049* (*batR*) at the *att*::*Tn7* insertion site, GmR	This study
Δ*PA0728* PAO1	PAO1 strain lacking the Pf4 integrase *PA0728*	[52]
*E. coli*		
DH5α	*endA*1, *hsdR*17(r_K_-m_K_+), *supE*44, *recA*1, *gyrA* (Nal^r^), *relA*1, Δ(*lacIZYA-argF*) U169, *deoR*, Φ80*dlacΔ(lacZ)M15*	[72]
BTH101	*F-, cya-99, araD139, galE15, galK16, rpsL1 (Strr), hsdR2, mcrA1, mcrB1.*	[46]
Plasmids		
pTS1	Tet^R^, suicide vector; *ColE1*-replicon, *IncP-1, Mob, lacZ*	[73]
pTS1-*batR* vector	pTS1 with Δ*batR* constructs as BamHI-HindIII inserts	This study
pME6032	Tet^R^, P_K_, 9.8 kb pVS1 derived shuttle vector	[74]
pUC18-mini-Tn*7*T-Gm	Amp^R^, Gm^R^, Tn*7* insertion vector	[75]
pTNS2	Helper plasmid for *att::*Tn*7* insertion	[75]
pME-*batR*	pME6032 containing the ORF of *batR* between the EcoRI-XhoI restriction sites	This study
pME-*srkA*	pME6032 containing the ORF of *srkA* (PA0486) between the EcoRI-XhoI restriction sites	This study
pME-*srkA* null	pME6032 containing the ORF of S33A D216A *srkA* (*srkA* null) between the EcoRI-XhoI restriction sites	This study
pUC18-mini-Tn*7*T-Gm-*batR*	pUC18-mini-Tn*7*T-Gm with *p*_*tac*_-*batR* fragment as a *Hind*III -*Spe*I insert.	This study
pMEXGFP	pMEX9 derived promoter-probe vector; *egfp* Gm^r^	[43]
pM0614-G	*PA0614* promoter region fused to *egfp* in pMEXGFP	[43]
pME-*batR-*3xFLAG	pME6032 containing the 3x-Flag-tagged *batR* ORF using EcoRI-XhoI restriction sites encoded in the outer primers (used in Co-IP assay)	This study
pKT25	Plasmid for constructing N-terminal fusions to T25, Kan^R^	[46]
pKT25-BatR	pKT25 with the ORF of *batR* cloned within the XbaI/KpnI sites (used in B2H assay)	This study
pKT25-AcpP	pKT25 with the ORF of *acpP* (*PA2966*) cloned within the XbaI/KpnI sites (used in B2H assay)	This study
pKT25-PslC	pKT25 with the ORF of *pslC* (*PA2233*) cloned within the XbaI/KpnI sites (used in B2H assay)	This study
pKT25-PilG	pKT25 with the ORF of *pilG* (*PA0408*) cloned within the XbaI/KpnI sites (used in B2H assay)	This study
pKT25-RibA	pKT25 with the ORF of *ribA* (*PA4047*) cloned within the XbaI/KpnI sites (used in B2H assay)	This study
pKT25-SrkA	pKT25 with the ORF of *srkA* (*PA0486*) cloned within the XbaI/KpnI sites (used in B2H assay)	This study
pKT25-GlpK	pKT25 with the ORF of *glpK* (*PA3582*) cloned within the XbaI/KpnI sites using Gibson assembly (B2H assay)	This study
pUT18C	Plasmid for constructing C-terminal fusions to T18, Carb^R^	[46]
pKT25-Zip	pKT25 carrying the leucine zipper of GCN4, Kan^R^ (used as positive control in B2H assay)	[46]
pUT18-zip	pUT18 carrying the leucine zipper of GCN4, Kan^R^ (used as positive control in B2H assay)	[46]

Carbenicillin (Carb) was used at 100 μg/mL, Kanamycin (Kan) at 50 μg/mL, Tetracycline (Tet) at 12.5 μg/mL for *E. coli* and 100 μg/mL for *P. aeruginosa*, IPTG at 0.5 mM, and X-gal at 40 μg/mL. The antibiotics Piperacillin (PIP), and Ciprofloxacin [16] were employed at concentrations optimized for each experiment, with details provided accordingly.

### Molecular biology techniques and genetic manipulation of PAO1

These procedures were performed as previously described [76]. All pTS1 plasmid inserts were synthesised and cloned into pTS1 by Twist Bioscience.

The ORF of *srkA* with S33 and D216 substituted by an Ala in the *srkA* null mutant was synthesised by Twist Bioscience. The ORFs of *batR* and *srkA* / *srkA* null were amplified by PCR with primers batR_EcoRI_F / batR_XhoI_R and srkA_EcoRI_F / srkA_XhoI_R, respectively (Table 6), and ligated between the EcoRI and XhoI sites of pME6032.

**Table 6:** Primers used in this study.

Primer name	Primer sequence*	Use	Plasmid cloned into
**Cloning**			
batR_EcoRI_F	TATGAATTCATGAGAAGACTTAAGCGTGATCC	Restriction Digest (EcoRI)	pME6032
batR_XhoI_R	TATCTCGAGTTACACGTGCTGGAGTTGATTGAG	Restriction Digest (XhoI)	pME6032
ptacFP	CGCGAAGCTTGACATCATAACGGTTCTG	Restriction Digest (HindIII)	pUC18-mini-*Tn7*T-Gm
ptacRP	CGCGACTAGTGTCCGAGGAGCTTTATGC	Restriction Digest (SpeI)	pUC18-mini-*Tn7*T-Gm
3xflag_batR_XhoI	TATCTCGAGTTTACTTGTCATCGTCGTCCTTGTAATCCTTGTCATCGTCGTCCTTGTAATCCTTGTCATCGTCGTCCTTGTAATCGCCGCCGCCCACGTGCTGGAGTTGATTGAG	Restriction Digest (XhoI) C_Flag tag Co_IP assay	pME6032
srkA-EcoRI-F	TATGAATTCATGTCCCATCCCTTCGACC	Restriction Digest (EcoRI)	pME6032
srkA-XhoI-R	TATCTCGAGTCAGAACAGCCGCAGCG	Restriction Digest (XhoI)	pME6032
**RT-qPCR**			
PA0629-RT-S	GTGGAGAACCTCAATTACAG	RT-qPCR	[77]
PA0629-RT-AS	TAGGTGTTGTCGGCAATC	RT-qPCR	[77]
Pf-Conserve-F	TTCCCGCGTGGAATGC	RT-qPCR	[78]
Pf-Conserve-R	CGGGAAGACAGCCACCAA	RT-qPCR	[78]
rpoD-F	CGCCAAGAAGTACACCAACC	RT-qPCR	This study
rpoD-R	GCGACGGTATTCGAACTTGT	RT-qPCR	This study
**Bacterial two hybrid**			
batR_XbaI_F	CAGATCTAGACATGAGAAGACTTAAGCGTGATCC	Restriction Digest	pKT25
batR_KpnI_R	ATATGGTACCTTACACGTGCTGGAGTTGATTGAG	Restriction Digest	pKT25 & pUT18C
batR_EcoRI_R	CAGAGAATTCGACACGTGCTGGAGTTGATTGAGAC	Restriction Digest	pUT18C
acpP_XbaI_F	CAGATCTAGACATGAGCACCATCGAAGAACG	Restriction Digest	pKT25
acpP1_KpnI_R	ATATGGTACCTTATTGCTGGTGAGCAACGATG	Restriction Digest	pKT25
pilG_XbaI_F	CAGATCTAGACATGGAACAGCAATCCGACG	Restriction Digest	pKT25
pilG_KpnI_R	ATATGGTACCTCAGGAAACGGCGTCCAC	Restriction Digest	pKT25
pslC_XbaI_F	CAGATCTAGACATGCGCTGCGCCCTG	Restriction Digest	pKT25
pslC_KpnI_R	ATATGGTACCTCACTTCCAGTAGCCTGGAAAc	Restriction Digest	pKT25
ribA_XbaI_F	CAGATCTAGACGTGTCCGTCGTGTTCGTC	Restriction Digest	pKT25
ribA_KpnI_R	ATATGGTACCTCACGTGGTTTCCGCCTC	Restriction Digest	pKT25
srkA_XbaI_F	CAGATCTAGACATGTCCCATCCCTTCGACC	Restriction Digest	pKT25
srkA_KpnI_R	ATATGGTACCTCAGAACAGCCGCAGCG	Restriction Digest	pKT25
glpK_XbaI_F	CAGATCTAGACATGACCGACAAGCACAACAAGAA	Restriction Digest	pKT25
glpK-EcoRI-R	CAGAGAATTCTCACAGTTCGCCGTCGTC	Restriction Digest	pKT25

* The restriction enzyme cutting sites are indicated in red, and the initiation codon ATG is underlined.

To introduce *batR* gene into the PAO1 *att*::*Tn7* site in the strain ∆*batR*c, the *batR* locus was amplified from the PAO1 genome using primers *batR*_EcoRI_F and *batR*_XhoI_R. The resulting PCR product was cloned into the multiple cloning site of pME6032 [74]. Primers ptacFP and ptacRP [76] were then used to amplify *batR* in addition to the *tac* promoter and terminator of pME6032. This product was then cloned into the pUC18-mini-*Tn7*T-Gm vector [75] and used to transform PAO1 via electroporation.

For the flag-tagged *batR* construction in pME6032, primers batR_EcoRI_F and 3xflag_batR_XhoI (Table 6) were used. Bacterial-2-hybrid fusion proteins were created by fusing the N-terminus of BatR, AcpP, SrkA, PslC, PilG, GlpK and RibA to pKT25 and combined with pUT18C-Empty and pUT18C-batR (Table 6). Genes were amplified using primers that introduce either XbaI-KpnI (stop codon) or XbaI-EcoRI (stop codon) restriction sites.

### MIC determination

Minimum inhibitory concentrations of antibiotics were determined by the broth microdilution method [37] following the EUCAST guidelines, using Mueller-Hinton broth. The Sub Inhibitory Concentration (SIC) was defined as being 1/8 of the lowest antibiotic concentration that inhibited visible growth after overnight incubation at 37°C.

### Inhibition disc assay

Bacterial cultures were grown in LB medium at 37°C to mid-log phase, A_600nm_=0.5-0.7, and 100 µL were spread on each plate. Whatmann filter paper discs containing the antibiotics were gently placed on the agar and plates were incubated inverted overnight. The normalized width of the antimicrobial “halo” (NW_halo_) of each disk was determined after [39].

### Glass beads biofilms

These assays were performed as described previously [38,79] with the following modifications. Ten independent biological replicates were included: five PIP-exposed biofilm lineages (challenged with SIC of PIP for 90 min) and five unexposed control lineages. Cells recovered from the beads were serially diluted and spotted onto LB plates for CFU counting. Results are shown as previously described by [38], where means in PIP-exposed biofilm were then normalised by the average number of cells across all unexposed conditions for plotting. Thus, the values represent the estimated proportion of cells that would survive each exposure for each strain. For complementation assays, culture media were supplemented with 0.5mM IPTG and Tet. The experiment was repeated three times.

### Membrane permeability assays

Differences in membrane permeability to antibiotics were assessed using the resazurin accumulation assay [80]. Strains of interest were grown to exponential phase under shaking conditions, using a 1:100 inoculum from overnight cultures. Cells were washed, resuspended in PBS, and normalized for cell density before being mixed with resazurin in round-bottom microtiter plates to a final volume of 100 µL (10 µg/mL resazurin). Fluorescence was measured with an Omega FLUOstar plate reader at an excitation wavelength of 544 nm and an emission wavelength of 590 nm. Five replicates were included per strain, and resazurin-only wells were used as controls. The assay was repeated at least twice, yielding reproducible results each time.

### Quantitative proteomics (TMT) for expression analysis

The experiment was essentially performed as previously described [81] with some modifications, as detailed in Supplementary Information (S4 Data).

### *PA0614* promoter activity assay

Promoter activity was assessed using the *egfp* reporter plasmid pMEXGFP [43]. Cells harbouring the recombinant plasmid were cultured on LB agar medium supplemented with Gn at 37 °C for 48 h. Following incubation, cells were scraped from the agar surface and resuspended in 1mL PBS. Fluorescence was measured using a fluorometer with an excitation wavelength of 488 nm and an emission wavelength of 509 nm. The eGFP fluorescence intensity of each sample was normalized against cells containing the promoterless pMEXGFP plasmid to account for background fluorescence.

### Time-lapse experiment using the CellASIC microfluidic system and image analysis

Time-lapse imaging of *P. aeruginosa* strains was performed as previously described [44], with the following modifications. Briefly, *P. aeruginosa* strains grown overnight in LB medium at 37°C and 250 rpm were sub-cultured to reach an OD_600_ of 0.2. Bacterial cells were loaded into B04A microfluidic plates (ONIX, CellASIC), grown by perfusing LB for 6 h in the case of PAO1 WT and ∆*batR* strains; or perfusing LB for 1 h, then switched to 0.1 mM IPTG LB for 9 h, and LB for 1h in the case of PAO1 strains overexpressing *srkA*. The media flow rate and temperature were maintained at 2 psi and 37°C. Time-lapse imaging was started from the beginning of the experiment and images were acquired every 5 min.

*P. aeruginosa* strains were visualized using a Zeiss Axio Observer Z.1 inverted epifluorescence microscope fitted with a sCMOS camera (Hamamatsu Orca FLASH 4), a Zeiss Colibri 7 LED light source, a Hamamatsu Orca Flash 4.0v3 sCMOS camera, and a temperature-controlled incubation chamber. Images were acquired using a Zeiss Plan Apochromat 100x/NA 1.4 Ph3 objective. Still images and time-lapse images series were collected using Zen Blue (Zeiss) and analysed using Fiji [82].

### Co-immunoprecipitation and mass spectrometry analysis

This protocol is detailed in Supplementary information (S5 Data).

### Bacterial 2 hybrid assays

These assays were performed as described elsewhere [81] with some modifications. The ORFs of *batR, acpP, pilG, pslC, ribA, srkA* and *glpK* were cloned into pKT25, and pUT18C using either conventional restriction enzyme cloning or Gibson assembly, as indicated in Table 6.

### Alphafold3 model predictions

Protein structures were predicted from amino acid sequences using the Alphafold3 server (alphafoldserver.com). For PAO1 BatR (AF_AFQ9HZF9F1) and *E. coli* Rmf (AF-P0AFW2-F1-v4), initially protein structures were retrieved from AlphaFoldDB with interactions modelled independently using the AlphaFold3 server. For interactions, both the bait (BatR WT) and target (SrkA) amino acid sequences were input into the sever and predictions were modelled using default settings. Models were ranked based on their respective PTM/iPTM score and the highest ranked score was taken forward for analysis. Structural analysis and alignments were conducted in Pymol.

### RNA purification and RT-qPCR

Four biological replicates of each strain were set up as described above. A single colony from each strain was picked and grown overnight in 10 mL LB medium at 37 °C, 250 rpm. The OD600 of these cultures was measured and 1 mL LB cultures were set up at OD600 = 0.2 and plated on LB agar and incubated at 37 °C for 48h. Cells were scraped from the agar surface and harvested for RNA extraction. Pellets were resuspended in 150 μL 10 mM Tris-HCl (pH 8) and mixed with 700 μL of ice cold RLT + BME (RLT buffer (Qiagen) supplemented with 1% β-mercaptoethanol) and cells were lysed using a Fastprep (MP Bio) using Lysis matrix B beads (MP Bio). Lysis matrix was removed by centrifugation (13,000 × g, 3 min) and the supernatant was added to 450 μL of ethanol. The supernatant was applied to an RNeasy column and RNA extraction was performed as per the manufacturer’s instruction including the on-column DNA digest. After extraction, a Turbo DNase (Promega) digest was performed as per the manufacturer’s instruction and total RNA yield was quantified using a Qubit RNA broad spectrum assay kit as per the manufacturer’s instructions.

Absolute quantification of target genes alongside the normalising housekeeping gene (*rpoD*) was performed using the provided CFX Maestro software. The relative quantity of the sample of interest was calculated using a calibration curve of serial dilutions of purified genomic DNA (CFX Maestro Software User Guide, Appendix A). The primers used are listed in Table 6. Relative qPCR units were defined as the ratio between the absolute levels of each gene and the housekeeping gene *rpoD.*

### Colony biofilm morphology assay

Agar plates for colony morphology experiments were prepared as previously described [83]. Briefly, a mixture of 1% agar and 1% tryptone was autoclaved and cooled to 60°C before 20 μg/ml Coomassie blue, 40 μg/ml Congo red, Tetracycline, and 0.05mM IPTG were added. For colony spotting, five microliters of cultures at an OD_600_ of 0.5 were spotted on plates and incubated for up to 7 days at 23–25°C.

### Cell viability and pyocyanin production phenotypes

Five biological replicates of each strain were grown in LB medium at 37°C to mid-log phase (A_600nm_=0.2-0.3). Then, 25 µL of each culture were spread onto wells of 24-well plates. When required, tetracycline and IPTG were added at the appropriate concentrations. Plates were incubated for 24h at 37°C and scanned. For cell viability measurements, cells were scraped from the agar and resuspended in 1mL of 1X PBS. These resuspensions were serially diluted and spotted onto LB agar plates for CFU enumeration.

### Crystal Violet (CV) assays

For the CV assay, five biological replicates of each strain were grown overnight in LB medium supplemented with Tet and then diluted into 200 μL of fresh LB to give an OD_600nm_ of 0.50 in microtiter plates. The expression of recombinant PAO1 *srkA*, was induced with 0.05mM IPTG. The microtiter plates were incubated at 37°C for 24 h, after which the wells were emptied and rinsed three times with water before staining. For staining, 200 μL of 0.1% CV was added to each well and incubated for 15 min at room temperature. The crystal violet dye was then removed, and the wells were rinsed with water. The dye bound to the cells was then dissolved in 70% ethanol, and the A_590nm_ was measured using a SPECTROstar nano plate reader (BMG Labtech).

### Genomic analysis

Six escape mutants -three from WT PAO1-pME-*srkA* null and three from ∆*batR* PAO1-pME-*srkA* null- were chosen for whole-genome sequencing. These colonies were re isolated from the colony biofilm morphology assay at day 7. As controls, the genomes of WT and ∆*batR* PAO1 strains were also sequenced. Whole-genome sequencing was performed by Plasmidsaurus using the Oxford Nanopore long-read technology. Variants were called against the ancestral reference genome using the Breseq computational pipeline using the polymorphic settings [84]. All variants were validated visually using the alignment viewer IGB [85].

### Bacteriophage experiments

Strains of interest were grown overnight in 10 mL of LB broth at 37°C. Cultures were adjusted to an OD_600_ of 1.0, and 100 µL of the normalized cultures were spread onto LB agar plates. Plates were incubated for 24 hours at 37°C and the resulting bacterial lawns were scraped from the plates and resuspended in 5 mL of 1 X PBS. These homogenates were passed through a 0.2 μm syringe filter to remove bacterial cells.

Indicator strains were grown overnight in 10 mL of LB broth and diluted 1,000-fold in PBS. Then, 50 µL of the diluted indicator cultures was spread evenly onto LB agar plates. Next, 10 µL of the filtered supernatants was spotted onto the indicator strain plates. Plates were incubated overnight at 37°C. As a control, filtered supernatants were spotted onto LB agar plates to confirm that they were cell-free.

### Label free proteomics

This protocol is detailed in Supplementary information (S6 Data)

### Synthetic chronic wound infection model

These assays were performed as previously described [34]. Briefly, batch cultures were grown at 37°C in Synthetic Wound Fluid (SWF) overnight to early mid log phase (approx. 6 h). The synthetic wounds were prepared as follows: For 10 mL collagen solution (2 mg/mL), 1 mL 0.1% acetic acid was mixed with 2 mL collagen stock solution (10 mg/mL) and kept on ice. Then, 6.0 mL of cold Synthetic Wound Fluid (SWF) (50% foetal bovine serum (Gibco 10270) and 50% Peptone water (Fluka 70179)) was added followed by 1 mL 0.1 M NaOH. After mixing, 200 μL of the collagen solution was added to each well (24-well plates). To achieve a complete polymerization of the collagen, the plates were placed in an incubator at 37°C for 1 h.

The starter bacterial cultures were diluted at an OD_600_ of approx. 0.05 – 0.1 in SWF. For small wounds, 50 μL of the diluted starter culture was added to each synthetic wound. Plates were incubated at 37°C for 24 h. After this time, 100 μL of antibiotic CIP at different concentrations was added to the wounds and plates were returned to 37°C for a further 24h. For bacterial recovery, 0.5 mg/mL collagenase (made in PBS) was added to each wound and incubated at 37°C for 1 h. Serial dilutions of these homogenates were plated on LB agar for CFU counting.

### EVPL infection model

EVPL was prepared as previously described [53,86]. Briefly, porcine lungs were obtained from two local butchers (Quigley and Sons, Cubbington and Taylor’s Butcher, Earlsdon) and dissected on the day of delivery under sterile conditions. The pleura of the ventral surface was heat sterilised using a hot pallet knife. A sterile razor blade was then used to make an incision in the lung, exposing the bronchiole. A section of the bronchiole was extracted, and the exterior alveolar tissue removed using dissection scissors. Bronchiolar sections were washed once in a 1:1 mix of Dulbecco’s modified Eagle medium (DMEM) and RPMI 1640 supplemented with 50 μg/mL ampicillin (Sigma-Aldrich) then cut into approximately 5 mm wide longitudinal strips. The bronchiolar strips were placed in a second 1:1 DMEM, RPMI 1640 supplemented with 50 μg/mL ampicillin wash and cut into squares approximately 5 x 5 mm in size. The tissue squares were washed for a third time in 1:1 DMEM, RPMI 1640 containing 50 μg/mL ampicillin. Bronchiolar pieces were then further washed in SCFM, UV sterilised for 5 min and transferred to individual wells of a 24-well plate containing 400 μL SCFM supplemented with 20 μg/mL ampicillin and solidified with 0.8% (w/v) agarose per well.

A sterile 29G hypodermic needle (Becton Dickinson Medical) was touched to the surface of a *P. aeruginosa* colony grown on LB agar overnight at 37°C and used to pierce an individual piece of bronchiolar tissue. Uninfected control tissue sections were mock inoculated with a fresh, sterile needle. Following infection of bronchiolar tissue pieces, 500 μL of SCFM ± 16 μg/mL CIP was added to each well. Tissue pieces were incubated at 37°C with a Breathe-Easier membrane (Diversified Biotech) for 2 and 7 d.

### EVPL biofilm recovery, assessment of bacterial load and pyocyanin production

EVPL biofilm recovery and assessment of bacterial load and virulence factor production were determined as described [53]. Briefly, bronchiolar tissue pieces were removed from the 24-well plate following incubation, and each briefly washed in 500 μL PBS in a fresh 24-well plate to remove planktonic cells. Tissue pieces were then transferred into sterile homogenisation tubes (Fisherbrand) containing eighteen 2.38 mm metal beads (Fisherbrand) and 1 mL PBS. Tissue was bead beaten in a FastPrep-24 5G (MP Biomedicals) for 40 s at 4 m/s to recover the bacteria and virulence factors from the tissue-associated biofilm. To determine the bacterial load, the homogenate was serially diluted in PBS and plated on LB agar. Plates were incubated overnight at 37°C and colony counts used to calculate colony forming units (CFU) per tissue piece.

To quantify pyocyanin produced by the *P. aeruginosa* biofilms, the homogenate was diluted 1:4 in PBS to obtain a sufficient volume for further experiments. The diluted homogenate was passed through a 0.2 μm filter to remove bacterial cells and tissue debris. Total pyocyanin was quantified by measuring absorbance of homogenates at 695 nm [33].

### Haematoxylin & eosin staining

*P. aeruginosa* infected EVPL tissue pieces and uninfected control tissue were fixed in 10% (v/v) neutral buffered formalin (VWR Chemicals), as previously described [53]. The infected/uninfected EVPL tissue pieces were fixed and sent to the University of Manchester’s Histology Core Facility for paraffin wax embedding, sectioning, and mounting. Mounted tissue sections were de-paraffinized in xylene for 20 min. To re-hydrate the tissue, slides were transferred to 95% (v/v) ethanol followed by 70% (v/v) ethanol. Any residual ethanol was removed by washing slides in distilled water. Samples were stained in Mayer’s hemalum solution (Merck Millipore) then washed in running tap water (1 L tap water with approximately 10 g sodium carbonate). Samples were counterstained in eosin Y solution (Merck Millipore) for then dehydrated by dipping the slides in 95% (v/v) ethanol. Samples were transferred to fresh 95% (v/v) ethanol then placed in 100% (v/v) ethanol. The samples were then placed in xylene. The samples were mounted using DPX mounting fluid and images were taken using a Zeiss Axio Imager Z2 light microscope with the Zeiss AxioCam 506 and Zeiss Zen Blue v2.3 pro software.

### Data presentation and statistical analyses

All graphs and statistical analyses, including one-way and two-way ANOVA followed by post-hoc Tukey's Multiple Comparison Test, when appropriate, were performed using GraphPad Prism version 5.04 for Windows.

### Data availability

The TMT proteomics data have been deposited to the ProteomeXchange Consortium via the PRIDE [87] partner repository with the dataset identifier PXD050997 and 10.6019/PXD050997.

The label-free proteomic data have been deposited to the ProteomeXchange Consortium via the PRIDE [85] partner repository with the dataset identifier PXD062857 and 10.6019/PXD062857.

Co-IP data are available via ProteomeXchange with identifier PXD050995.

## Supporting information

S1 Data Integrated Quantitative (TMT) Proteomic data comparing WT and ∆*batR* PAO1 strains. Contains underlying data for Tables 1 and 2.
(XLSX)

S2 Data BatR interacting proteins (Co-immunoprecipitation assay). Contains underlying data for Table 3 and Fig 3.
(XLSX)

S3 Data Label-free Proteomics comparing WT and ∆*batR* PAO1 supernatants. Contains underlying data for Table 4.
(XLSX)

S4 Data Quantitative Proteomics (TMT) expression analysis. (DOCX)

S5 Data Co-Immunoprecipitation and mass spectrometry analysis. (DOCX)

S6 Data Label-free Proteomics analysis. (DOCX)

S1 Fig Growth curves are shown for the following strains a WT pME-Empty, Δ*batR* pME-Empty and Δ*batR* pME-*batR*; b WT pME-Empty, Δ*batR* pME-Empty, WT pME-*srkA*, Δ*batR* pME-*srkA,* WT pME-*srkA* (null) and Δ*batR* pME-*srkA* (null); and c WT, Δ*batR* and Δ*batR*c grown in LB medium. Where indicated, cultures were supplemented with tetracycline and IPTG. Mean growth from 5 biological replicates is shown as a solid line, with standard deviation indicated as dotted lines. Cells were grown for 24 h at 37 °C under shaking conditions.
(DOCX)

S2 Fig BatR does not affect membrane permeability. Drug accumulation was assessed by measuring resazurin fluorescence at an excitation wavelength of 544 nm and an emission wavelength of 590 nm (544/590 nm) for 2 hours (a), 12 hours (b) and 18 hours (c). Lines represent the mean of three biological replicates, each with four technical replicates, with the error bars showing the standard deviation. No significant differences were observed between the strains tested.
(DOCX)

S3 Fig a. **Genetic organization of the R2 pyocin gene locus in *P. aeruginosa* PAO1.** Structure of the R2-F2 pyocin gene cluster in *P. aeruginosa* PAO1. Grey indicates regulatory genes; yellow, the lysis cassette; green, R2-type pyocin genes; and orange, F2-type pyocin genes. The function of individual proteins is labelled, and proteins enriched in the WT strain proteome are boxed. Gene sizes are drawn to scale. **b. Effect of BatR on pyocin production.** The effect of BatR on the promoter activity of *PA0614* was measured using pME0614-G, normalised to pMEXGFP. **c. Proportion of cells exhibiting a round-cell morphotype in microfluidics assays.** Cells were classified as either rod-shaped or round over a 3-hour period, and the frequency of round cells was calculated as the number of round cells divided by the total number of cells counted at *t* = 180 min. Results were analysed by a one-way ANOVA showing no significant differences between WT and Δ*batR* strains under the conditions tested. **d.** Phase-contrast time-lapse images showing rod-to-round cell transition and explosive lysis events (highlighted by red circles) in WT and Δ*batR* strains. Time is indicated in minutes (top right); scale bar, 2 μm.
(DOCX)

S4 Fig Phenotypes of PAO1 WT; ∆*batR;* ∆*srkA;* and ∆*batR* ∆*srkA* strains. **a. Biofilm formation.** Strains were grown statically in LB medium for 24h at 37°C. Biofilm biomass was quantified by Crystal Violet staining and measured spectrophotometrically at 590 nm (A_590nm_.). Values represent the mean of five biological replicates with two technical replicates each; error bars indicate SD. **b. Cell viability.** Cells were scraped from LB agar plates, resuspended in PBS, and enumerated via serial dilution and plating. **c. Pyocyanin production.** Top view images of cell lawns showing pyocyanin production (blue colouration). **d. Growth curve.** Growth curves are shown for strains WT (blue), Δ*batR* (orange), ΔsrkA (gray); and Δ*batR* ΔsrkA (yellow)in LB medium. The mean growth for 6 biological replicates is shown as a solid line and standard deviation shown as dotted lines. Cells were grown for 48 h at 37 °C under shaking conditions. **e, f.** Influence of *batR* and *srkA* deletions on *PA0629* (*lys*) and *PA0727* (*Pf4*) transcripts, respectively as determined by qRT-PCR. Transcript levels were normalised against *rpoD*. One-way ANOVA with Tukey's multiple comparisons was used to compare means, p < 0.0001 ***.
(DOCX)

S5 Fig Overexpression of *srkA* null activates prophage Pf4 in *P. aeruginosa.* The depth graph shows the distribution of sequencing read coverage across the genomic region spanning the Pf4 cluster (PA0717-PA0727), with each gene represented by a blue arrow. Three PAO1 WT-derived mutants overexpressing *srkA* null are shown.
(DOCX)

S6 Fig MIC of PAO1 wild type in the EVPL Model. Log-transformed total CFU of *P. aeruginosa* PAO1 WT recovered from the EVPL model following treatment with different concentrations of CIP. Three biological replicates were grown on EVPL tissue for 48 h, then exposed to CIP or PBS as a control for 18 h. CFU/lung was determined post treatment. The MIC of CIP in this model was 128 µg/mL.
(DOCX)

S7 Fig Haematoxylin and eosin (H & E) stained sections of EVPL bronchiolar tissue with SCFM medium infected with *P. aeruginosa* at 2 d post infection. EVPL was infected with *P. aeruginosa* PAO1 WT and ∆*batR*, with uninfected tissue as a negative control. The x20 magnification images from the sections are shown here for non-treated tissues (SCFM) and treated with CIP (SCFM + CIP). Representative images of phenotypes at 2dpi are shown here, but the same results were observed for all biological replicates analysed.
(DOCX)

## References

[1] Rice LB. Federal funding for the study of antimicrobial resistance in nosocomial pathogens: no ESKAPE. J Infect Dis. 2008;197(8):1079–81. doi: 10.1086/533452 18419525

[2] Shigemura K, Arakawa S, Sakai Y, Kinoshita S, Tanaka K, Fujisawa M. Complicated urinary tract infection caused by Pseudomonas aeruginosa in a single institution (1999-2003). Int J Urol. 2006;13(5):538–42. 16771722 10.1111/j.1442-2042.2006.01359.x

[3] Chitkara YK, Feierabend TC. Endogenous and exogenous infection with Pseudomonas aeruginosa in a burns unit. Int Surg. 1981;66(3):237–40. 6797982

[4] Mulcahy LR, Isabella VM, Lewis K. Pseudomonas aeruginosa biofilms in disease. Microb Ecol. 2014;68(1):1–12. doi: 10.1007/s00248-013-0297-x 24096885 PMC3977026

[5] Kielhofner M, Atmar RL, Hamill RJ, Musher DM. Life-threatening Pseudomonas aeruginosa infections in patients with human immunodeficiency virus infection. Clin Infect Dis. 1992;14(2):403–11. doi: 10.1093/clinids/14.2.403 1554824

[6] Lyczak JB, Cannon CL, Pier GB. Lung infections associated with cystic fibrosis. Clin Microbiol Rev. 2002;15(2):194–222. doi: 10.1128/CMR.15.2.194-222.2002 11932230 PMC118069

[7] Pressler T, Bohmova C, Conway S, Dumcius S, Hjelte L, Høiby N, et al. Chronic Pseudomonas aeruginosa infection definition: EuroCareCF Working Group report. J Cyst Fibros. 2011;10 Suppl 2:S75-8. doi: 10.1016/S1569-1993(11)60011-8 21658646

[8] Boyd CD, O’Toole GA. Second messenger regulation of biofilm formation: breakthroughs in understanding c-di-GMP effector systems. Annu Rev Cell Dev Biol. 2012;28:439–62. doi: 10.1146/annurev-cellbio-101011-155705 23057745 PMC4936406

[9] Serra DO, Hengge R. Stress responses go three dimensional - the spatial order of physiological differentiation in bacterial macrocolony biofilms. Environ Microbiol. 2014;16(6):1455–71. doi: 10.1111/1462-2920.12483 24725389 PMC4238805

[10] Piazza A, Parra L, Ciancio Casalini L, Sisti F, Fernández J, Malone JG, et al. Cyclic di-GMP Signaling Links Biofilm Formation and Mn(II) Oxidation in Pseudomonas resinovorans. mBio. 2022;13(6):e0273422. doi: 10.1128/mbio.02734-22 36374078 PMC9765421

[11] Williamson KS, Richards LA, Perez-Osorio AC, Pitts B, McInnerney K, Stewart PS, et al. Heterogeneity in Pseudomonas aeruginosa biofilms includes expression of ribosome hibernation factors in the antibiotic-tolerant subpopulation and hypoxia-induced stress response in the metabolically active population. J Bacteriol. 2012;194(8):2062–73. doi: 10.1128/JB.00022-12 22343293 PMC3318454

[12] Fajardo A, Martínez JL. Antibiotics as signals that trigger specific bacterial responses. Curr Opin Microbiol. 2008;11(2):161–7. doi: 10.1016/j.mib.2008.02.006 18373943

[13] Davies J, Spiegelman GB, Yim G. The world of subinhibitory antibiotic concentrations. Curr Opin Microbiol. 2006;9(5):445–53. doi: 10.1016/j.mib.2006.08.006 16942902

[14] Ranieri MR, Whitchurch CB, Burrows LL. Mechanisms of biofilm stimulation by subinhibitory concentrations of antimicrobials. Curr Opin Microbiol. 2018;45:164–9. doi: 10.1016/j.mib.2018.07.006 30053750

[15] Wright EA, Fothergill JL, Paterson S, Brockhurst MA, Winstanley C. Sub-inhibitory concentrations of some antibiotics can drive diversification of populations in artificial sputum medium. BMC Microbiol. 2013;13. 10.1186/1471-2180-13-170 PMC3726342 23879797

[16] Taccetti G, Francalanci M, Pizzamiglio G, Messore B, Carnovale V, Cimino G. Cystic Fibrosis: Recent Insights into Inhaled Antibiotic Treatment and Future Perspectives. Antibiotics-Basel. 2021;10(3). 10.3390/antibiotics10030338 PMC8004710 33810116

[17] Dwyer DJ, Belenky PA, Yang JH, MacDonald IC, Martell JD, Takahashi N, et al. Antibiotics induce redox-related physiological alterations as part of their lethality. Proc Natl Acad Sci U S A. 2014;111(20):E2100-9. doi: 10.1073/pnas.1401876111 24803433 PMC4034191

[18] Linares JF, Gustafsson I, Baquero F, Martinez JL. Antibiotics as intermicrobial signaling agents instead of weapons. Proc Natl Acad Sci U S A. 2006;103(51):19484–9. doi: 10.1073/pnas.0608949103 17148599 PMC1682013

[19] Nalca Y, Jänsch L, Bredenbruch F, Geffers R, Buer J, Häussler S. Quorum-sensing antagonistic activities of azithromycin in Pseudomonas aeruginosa PAO1: a global approach. Antimicrob Agents Chemother. 2006;50(5):1680–8. doi: 10.1128/AAC.50.5.1680-1688.2006 16641435 PMC1472232

[20] Hoffman LR, D’Argenio DA, MacCoss MJ, Zhang Z, Jones RA, Miller SI. Aminoglycoside antibiotics induce bacterial biofilm formation. Nature. 2005;436(7054):1171–5. doi: 10.1038/nature03912 16121184

[21] Kaplan JB. Antibiotic-induced biofilm formation. Int J Artif Organs. 2011;34(9):737–51. doi: 10.5301/ijao.5000027 22094552

[22] Whitchurch CB, Tolker-Nielsen T, Ragas PC, Mattick JS. Extracellular DNA required for bacterial biofilm formation. Science. 2002;295(5559):1487. doi: 10.1126/science.295.5559.1487 11859186

[23] Turnbull L, Toyofuku M, Hynen AL, Kurosawa M, Pessi G, Petty NK, et al. Explosive cell lysis as a mechanism for the biogenesis of bacterial membrane vesicles and biofilms. Nat Commun. 2016;7:11220. doi: 10.1038/ncomms11220 27075392 PMC4834629

[24] Vacheron J, Heiman CM, Keel C. Live cell dynamics of production, explosive release and killing activity of phage tail-like weapons for kin exclusion. Commun Biol. 2021;4(1). 10.1038/s42003-020-01581-1 PMC7815802 33469108

[25] Petrova OE, Schurr JR, Schurr MJ, Sauer K. The novel Pseudomonas aeruginosa two-component regulator BfmR controls bacteriophage-mediated lysis and DNA release during biofilm development through PhdA. Mol Microbiol. 2011;81(3):767–83. doi: 10.1111/j.1365-2958.2011.07733.x 21696457 PMC3214647

[26] Allesen-Holm M, Barken KB, Yang L, Klausen M, Webb JS, Kjelleberg S, et al. A characterization of DNA release in Pseudomonas aeruginosa cultures and biofilms. Mol Microbiol. 2006;59(4):1114–28. doi: 10.1111/j.1365-2958.2005.05008.x 16430688

[27] Das T, Manefield M. Pyocyanin promotes extracellular DNA release in. PLoS One. 2012;7(10). 10.1371/journal.pone.0046718 PMC3466280 23056420

[28] Wilson R, Sykes DA, Watson D, Rutman A, Taylor GW, Cole PJ. Measurement of Pseudomonas aeruginosa phenazine pigments in sputum and assessment of their contribution to sputum sol toxicity for respiratory epithelium. Infect Immun. 1988;56(9):2515–7. doi: 10.1128/iai.56.9.2515-2517.1988 3137173 PMC259599

[29] Kaleta MF, Petrova OE, Zampaloni C, Garcia-Alcalde F, Parker M, Sauer K. A previously uncharacterized gene, PA2146, contributes to biofilm formation and drug tolerance across the ɣ-Proteobacteria. NPJ Biofilms Microbiomes. 2022;8(1):54. doi: 10.1038/s41522-022-00314-y 35798749 PMC9262955

[30] Yamagishi M, Matsushima H, Wada A, Sakagami M, Fujita N, Ishihama A. Regulation of the Escherichia coli rmf gene encoding the ribosome modulation factor: growth phase- and growth rate-dependent control. EMBO J. 1993;12(2):625–30. doi: 10.1002/j.1460-2075.1993.tb05695.x 8440252 PMC413246

[31] Wada A, Yamazaki Y, Fujita N, Ishihama A. Structure and probable genetic location of a “ribosome modulation factor” associated with 100S ribosomes in stationary-phase Escherichia coli cells. Proc Natl Acad Sci U S A. 1990;87(7):2657–61. doi: 10.1073/pnas.87.7.2657 2181444 PMC53749

[32] Akiyama T, Williamson KS, Schaefer R, Pratt S, Chang CB, Franklin MJ. Resuscitation of Pseudomonas aeruginosa from dormancy requires hibernation promoting factor (PA4463) for ribosome preservation. Proc Natl Acad Sci U S A. 2017;114(12):3204–9. doi: 10.1073/pnas.1700695114 28270601 PMC5373351

[33] Harrison F, Muruli A, Higgins S, Diggle SP. Development of an ex vivo porcine lung model for studying growth, virulence, and signaling of Pseudomonas aeruginosa. Infect Immun. 2014;82(8):3312–23. doi: 10.1128/IAI.01554-14 24866798 PMC4136229

[34] Werthén M, Henriksson L, Jensen PØ, Sternberg C, Givskov M, Bjarnsholt T. An in vitro model of bacterial infections in wounds and other soft tissues. APMIS. 2010;118(2):156–64. doi: 10.1111/j.1600-0463.2009.02580.x 20132180

[35] Abramson J, Adler J, Dunger J, Evans R, Green T, Pritzel A, et al. Accurate structure prediction of biomolecular interactions with AlphaFold 3. Nature. 2024;630(8016):493–500. doi: 10.1038/s41586-024-07487-w 38718835 PMC11168924

[36] Prossliner T, Skovbo Winther K, Sorensen MA, Gerdes K. Ribosome hibernation. Annu Rev Genet. 2018;52:321–48. 30476446 10.1146/annurev-genet-120215-035130

[37] Susc ECA. Determination of minimum inhibitory concentrations (MICs) of antibacterial agents by agar dilution. Clin Microbiol Infec. 2000;6(9):509–15. 11168187 10.1046/j.1469-0691.2000.00142.x

[38] Trampari E, Holden ER, Wickham GJ, Ravi A, Martins L de O, Savva GM, et al. Exposure of Salmonella biofilms to antibiotic concentrations rapidly selects resistance with collateral tradeoffs. NPJ Biofilms Microbiomes. 2021;7(1):3. doi: 10.1038/s41522-020-00178-0 33431848 PMC7801651

[39] Marti M, Frigols B, Serrano-Aroca A. Antimicrobial characterization of advanced materials for bioengineering applications. J Vis Exp. 2018;138. 10.3791/57710 PMC6126623 30124638

[40] Reales-Calderón JA, Sun Z, Mascaraque V, Pérez-Navarro E, Vialás V, Deutsch EW, et al. A wide-ranging Pseudomonas aeruginosa PeptideAtlas build: A useful proteomic resource for a versatile pathogen. J Proteomics. 2021;239:104192. doi: 10.1016/j.jprot.2021.104192 33757883 PMC8668395

[41] Nakayama K, Takashima K, Ishihara H, Shinomiya T, Kageyama M, Kanaya S, et al. The R-type pyocin of Pseudomonas aeruginosa is related to P2 phage, and the F-type is related to lambda phage. Mol Microbiol. 2000;38(2):213–31. doi: 10.1046/j.1365-2958.2000.02135.x 11069649

[42] Toyofuku M, Nomura N, Eberl L. Types and origins of bacterial membrane vesicles. Nat Rev Microbiol. 2019;17(1):13–24. doi: 10.1038/s41579-018-0112-2 30397270

[43] Toyofuku M, Zhou S, Sawada I, Takaya N, Uchiyama H, Nomura N. Membrane vesicle formation is associated with pyocin production under denitrifying conditions in Pseudomonas aeruginosa PAO1. Environ Microbiol. 2014;16(9):2927–38. doi: 10.1111/1462-2920.12260 24112564

[44] Schlimpert S, Flardh K, Buttner J. Fluorescence time-lapse imaging of the complete S. venezuelae life cycle using a microfluidic device. J Vis Exp. 2016;108:53863. 10.3791/53863 PMC4828195 26967231

[45] Dorsey-Oresto A, Lu T, Mosel M, Wang X, Salz T, Drlica K, et al. YihE kinase is a central regulator of programmed cell death in bacteria. Cell Rep. 2013;3(2):528–37. doi: 10.1016/j.celrep.2013.01.026 23416055 PMC3594398

[46] Karimova G, Pidoux J, Ullmann A, Ladant D. A bacterial two-hybrid system based on a reconstituted signal transduction pathway. Proc Natl Acad Sci U S A. 1998;95(10):5752–6. doi: 10.1073/pnas.95.10.5752 9576956 PMC20451

[47] Zheng J, He C, Singh VK, Martin NL, Jia Z. Crystal structure of a novel prokaryotic Ser/Thr kinase and its implication in the Cpx stress response pathway. Mol Microbiol. 2007;63(5):1360–71. doi: 10.1111/j.1365-2958.2007.05611.x 17302814

[48] Qin S, Xiao W, Zhou C, Pu Q, Deng X, Lan L, et al. Pseudomonas aeruginosa: pathogenesis, virulence factors, antibiotic resistance, interaction with host, technology advances and emerging therapeutics. Signal Transduct Target Ther. 2022;7(1):199. doi: 10.1038/s41392-022-01056-1 35752612 PMC9233671

[49] Webb JS, Thompson LS, James S, Charlton T, Tolker-Nielsen T, Koch B, et al. Cell death in Pseudomonas aeruginosa biofilm development. J Bacteriol. 2003;185(15):4585–92. doi: 10.1128/JB.185.15.4585-4592.2003 12867469 PMC165772

[50] Rice SA, Tan CH, Mikkelsen PJ, Kung V, Woo J, Tay M, et al. The biofilm life cycle and virulence of Pseudomonas aeruginosa are dependent on a filamentous prophage. ISME J. 2009;3(3):271–82. doi: 10.1038/ismej.2008.109 19005496 PMC2648530

[51] Tortuel D, Tahrioui A, Rodrigues S, Cambronel M, Boukerb AM, Maillot O, et al. Activation of the Cell Wall Stress Response in Pseudomonas aeruginosa Infected by a Pf4 Phage Variant. Microorganisms. 2020;8(11):1700. doi: 10.3390/microorganisms8111700 33143386 PMC7693463

[52] Tarafder AK, Graham M, Davis LK, Pratt S, Bohning J, Manivannan P. Targeted disruption of phage liquid crystalline droplets abolishes antibiotic tolerance of bacterial biofilms. bioRxiv. 2025.

[53] Harrington NE, Sweeney E, Harrison F. Building a better biofilm - Formation of in vivo-like biofilm structures by Pseudomonas aeruginosa in a porcine model of cystic fibrosis lung infection. Biofilm. 2020;2:100024. doi: 10.1016/j.bioflm.2020.100024 33381751 PMC7762787

[54] Bjarnsholt T, Jensen PØ, Fiandaca MJ, Pedersen J, Hansen CR, Andersen CB, et al. Pseudomonas aeruginosa biofilms in the respiratory tract of cystic fibrosis patients. Pediatr Pulmonol. 2009;44(6):547–58. doi: 10.1002/ppul.21011 19418571

[55] Henderson AG, Ehre C, Button B, Abdullah LH, Cai L-H, Leigh MW, et al. Cystic fibrosis airway secretions exhibit mucin hyperconcentration and increased osmotic pressure. J Clin Invest. 2014;124(7):3047–60. doi: 10.1172/JCI73469 24892808 PMC4072023

[56] Baltimore RS, Christie CD, Smith GJ. Immunohistopathologic localization of Pseudomonas aeruginosa in lungs from patients with cystic fibrosis. Implications for the pathogenesis of progressive lung deterioration. Am Rev Respir Dis. 1989;140(6):1650–61. doi: 10.1164/ajrccm/140.6.1650 2513765

[57] Bielecki P, Puchałka J, Wos-Oxley ML, Loessner H, Glik J, Kawecki M, et al. In-vivo expression profiling of Pseudomonas aeruginosa infections reveals niche-specific and strain-independent transcriptional programs. PLoS One. 2011;6(9):e24235. doi: 10.1371/journal.pone.0024235 21931663 PMC3171414

[58] Serra DO, Richter AM, Klauck G, Mika F, Hengge R. Microanatomy at cellular resolution and spatial order of physiological differentiation in a bacterial biofilm. mBio. 2013;4(2):e00103-13. doi: 10.1128/mBio.00103-13 23512962 PMC3604763

[59] Ramos I, Dietrich LEP, Price-Whelan A, Newman DK. Phenazines affect biofilm formation by Pseudomonas aeruginosa in similar ways at various scales. Res Microbiol. 2010;161(3):187–91. doi: 10.1016/j.resmic.2010.01.003 20123017 PMC2886020

[60] Price-Whelan A, Dietrich LEP, Newman DK. Rethinking “secondary” metabolism: physiological roles for phenazine antibiotics. Nat Chem Biol. 2006;2(2):71–8. doi: 10.1038/nchembio764 16421586

[61] Das T, Manefield M. Pyocyanin promotes extracellular DNA release in Pseudomonas aeruginosa. PLoS One. 2012;7(10):e46718. doi: 10.1371/journal.pone.0046718 23056420 PMC3466280

[62] Das T, Kutty SK, Kumar N, Manefield M. Pyocyanin facilitates extracellular DNA binding to Pseudomonas aeruginosa influencing cell surface properties and aggregation. PLoS One. 2013;8(3):e58299. doi: 10.1371/journal.pone.0058299 23505483 PMC3594310

[63] Dietrich LEP, Teal TK, Price-Whelan A, Newman DK. Redox-active antibiotics control gene expression and community behavior in divergent bacteria. Science. 2008;321(5893):1203–6. doi: 10.1126/science.1160619 18755976 PMC2745639

[64] Dietrich LEP, Okegbe C, Price-Whelan A, Sakhtah H, Hunter RC, Newman DK. Bacterial community morphogenesis is intimately linked to the intracellular redox state. J Bacteriol. 2013;195(7):1371–80. doi: 10.1128/JB.02273-12 23292774 PMC3624522

[65] Schiessl KT, Hu F, Jo J, Nazia SZ, Wang B, Price-Whelan A, et al. Phenazine production promotes antibiotic tolerance and metabolic heterogeneity in Pseudomonas aeruginosa biofilms. Nat Commun. 2019;10(1):762. doi: 10.1038/s41467-019-08733-w 30770834 PMC6377615

[66] Garay-Arroyo A, Colmenero-Flores JM, Garciarrubio A, Covarrubias AA. Highly hydrophilic proteins in prokaryotes and eukaryotes are common during conditions of water deficit. J Biol Chem. 2000;275(8):5668–74. doi: 10.1074/jbc.275.8.5668 10681550

[67] Ameisen JC. On the origin, evolution, and nature of programmed cell death: a timeline of four billion years. Cell Death Differ. 2002;9(4):367–93. doi: 10.1038/sj.cdd.4400950 11965491

[68] Rice KC, Bayles KW. Death’s toolbox: examining the molecular components of bacterial programmed cell death. Mol Microbiol. 2003;50(3):729–38. doi: 10.1046/j.1365-2958.2003.t01-1-03720.x 14617136

[69] Dietrich LEP, Okegbe C, Price-Whelan A, Sakhtah H, Hunter RC, Newman DK. Bacterial community morphogenesis is intimately linked to the intracellular redox state. J Bacteriol. 2013;195(7):1371–80. doi: 10.1128/JB.02273-12 23292774 PMC3624522

[70] Miller JH. Experiments in molecular genetics. Cold Spring Harbor, N.Y.: Cold Spring Harbor Laboratory; 1972. xvi, 466 p.

[71] Holloway BW. Genetic recombination in Pseudomonas aeruginosa. J Gen Microbiol. 1955;13(3):572–81. doi: 10.1099/00221287-13-3-572 13278508

[72] Woodcock DM, Crowther PJ, Doherty J, Jefferson S, DeCruz E, Noyer-Weidner M, et al. Quantitative evaluation of Escherichia coli host strains for tolerance to cytosine methylation in plasmid and phage recombinants. Nucleic Acids Res. 1989;17(9):3469–78. doi: 10.1093/nar/17.9.3469 2657660 PMC317789

[73] Scott TA, Heine D, Qin Z, Wilkinson B. An L-threonine transaldolase is required for L-threo-β-hydroxy-α-amino acid assembly during obafluorin biosynthesis. Nat Commun. 2017;8:15935. doi: 10.1038/ncomms15935 28649989 PMC5490192

[74] Heeb S, Itoh Y, Nishijyo T, Schnider U, Keel C, Wade J, et al. Small, stable shuttle vectors based on the minimal pVS1 replicon for use in gram-negative, plant-associated bacteria. Mol Plant Microbe Interact. 2000;13(2):232–7. doi: 10.1094/MPMI.2000.13.2.232 10659714

[75] Choi K-H, Gaynor JB, White KG, Lopez C, Bosio CM, Karkhoff-Schweizer RR, et al. A Tn7-based broad-range bacterial cloning and expression system. Nat Methods. 2005;2(6):443–8. doi: 10.1038/nmeth765 15908923

[76] Woodcock SD, Syson K, Little RH, Ward D, Sifouna D, Brown JKM, et al. Trehalose and α-glucan mediate distinct abiotic stress responses in Pseudomonas aeruginosa. PLoS Genet. 2021;17(4):e1009524. doi: 10.1371/journal.pgen.1009524 33872310 PMC8084333

[77] Chen F, Chen G, Liu Y, Jin Y, Cheng Z, Liu Y, et al. Pseudomonas aeruginosa Oligoribonuclease Contributes to Tolerance to Ciprofloxacin by Regulating Pyocin Biosynthesis. Antimicrob Agents Chemother. 2017;61(3):e02256-16. doi: 10.1128/AAC.02256-16 28052848 PMC5328516

[78] Burgener EB, Secor PR, Tracy MC, Sweere JM, Bik EM, Milla CE, et al. Methods for Extraction and Detection of Pf Bacteriophage DNA from the Sputum of Patients with Cystic Fibrosis. Phage (New Rochelle). 2020;1(2):100–8. doi: 10.1089/phage.2020.0003 32626852 PMC7327540

[79] Piazza A, Ciancio Casalini L, Pacini VA, Sanguinetti G, Ottado J, Gottig N. Environmental Bacteria Involved in Manganese(II) Oxidation and Removal From Groundwater. Front Microbiol. 2019;10:119. doi: 10.3389/fmicb.2019.00119 30853942 PMC6396730

[80] Trampari E, Zhang C, Gotts K, Savva GM, Bavro VN, Webber M. Cefotaxime Exposure Selects Mutations within the CA-Domain of envZ Which Promote Antibiotic Resistance but Repress Biofilm Formation in Salmonella. Microbiol Spectr. 2022;10(3):e0214521. doi: 10.1128/spectrum.02145-21 35475640 PMC9241649

[81] Thompson CMA, Hall JPJ, Chandra G, Martins C, Saalbach G, Panturat S, et al. Plasmids manipulate bacterial behaviour through translational regulatory crosstalk. PLoS Biol. 2023;21(2):e3001988. doi: 10.1371/journal.pbio.3001988 36787297 PMC9928087

[82] Schindelin J, Arganda-Carreras I, Frise E, Kaynig V, Longair M, Pietzsch T, et al. Fiji: an open-source platform for biological-image analysis. Nat Methods. 2012;9(7):676–82. doi: 10.1038/nmeth.2019 22743772 PMC3855844

[83] Cornell WC, Morgan CJ, Koyama L, Sakhtah H, Mansfield JH, Dietrich LEP. Paraffin embedding and thin sectioning of microbial colony biofilms for microscopic analysis. J Vis Exp. 2018;2018(133). 10.3791/57196 PMC5933251 29630036

[84] Deatherage DE, Barrick JE. Identification of mutations in laboratory-evolved microbes from next-generation sequencing data using breseq. Methods Mol Biol. 2014;1151:165–88. doi: 10.1007/978-1-4939-0554-6_12 24838886 PMC4239701

[85] Robinson JT, Thorvaldsdóttir H, Winckler W, Guttman M, Lander ES, Getz G, et al. Integrative genomics viewer. Nat Biotechnol. 2011;29(1):24–6. doi: 10.1038/nbt.1754 21221095 PMC3346182

[86] Harrington NE, Sweeney E, Alav I, Allen F, Moat J, Harrison F. Antibiotic Efficacy Testing in an Ex vivo Model of Pseudomonas aeruginosa and Staphylococcus aureus Biofilms in the Cystic Fibrosis Lung. J Vis Exp. 2021;167. 10.3791/62187 33554970

[87] Perez-Riverol Y, Bai J, Bandla C, García-Seisdedos D, Hewapathirana S, Kamatchinathan S, et al. The PRIDE database resources in 2022: a hub for mass spectrometry-based proteomics evidences. Nucleic Acids Res. 2022;50(D1):D543–52. doi: 10.1093/nar/gkab1038 34723319 PMC8728295

