# Supplementary material for: A novel stress response pathway mediates biofilm architecture in Pseudomonas aeruginosa
Source: PLoS Pathog. 2026 Jul 28;22(7):e1013832. doi: 10.1371/journal.ppat.1013832 (PMC13411936; doi:10.1371/journal.ppat.1013832)
Supplement: S4 Data — (DOCX) [file ppat.1013832.s004.docx]

**S4 data. Quantitative Proteomics (TMT) expression analysis.**

Lawns of PAO1 strains were grown for 24h on LB agar plates, with four biological replicates each. The cells were scraped and resuspended in 1.5 mL cell lysis buffer (50 mM TEAB Buffer pH 8.0; 150 mM NaCl; 2% SDS; EDTA-free protease inhibitor (Roche)). The suspension was boiled for 10 min and then sonicated at 8 mA four times for 20 s per cycle and then pelleted at 4,000 x g for 30 min. Protein concentration was determined using the BCA assay and 2 mg of protein from each sample transferred to a fresh 15 ml Falcon tube. The proteins were precipitated with chloroform/methanol according to [1]. The resulting protein pellets were washed with acetone and then resuspended in 200 µl of 1.25% sodium deoxycholate (SDC; Merck) in 0.2 M EPPS-buffer (Merck), pH 8.5, and vortexed under heating. Protein concentration was estimated using a BCA assay. Using approx. 100 µg of protein per sample, cysteine residues were reduced with dithiothreitol, alkylated with iodoacetamide, and the proteins digested with trypsin in the SDC buffer according to standard procedures. After the digest, the SDC was precipitated by adjusting to 0.2% trifluoroacetic acid (TFA), and the clear supernatant subjected to C18 SPE (Reprosil, Dr. Maisch GmbH, Germany). Peptide concentration was further estimated by running an aliquot of the digests on LCMS (as described below). Tandem Mass Tag (TMT) labelling was performed using a TMT™16plex kit (Lot WJ329096, ThermoFisher Scientific, Hemel Hempstead, UK) according to the manufacturer’s instructions with slight modifications; the dried peptides were dissolved in 90 µl of 0.2 M EPPS buffer (Merck)/10% acetonitrile, and 250 µg TMT reagent dissolved in 22 µl of acetonitrile was added. Samples were assigned to the TMT channels in an order avoiding channel leakage between different conditions if possible (see Brenes et al., Mol Cell Proteomics. 2019 Oct;18(10):1967-1980.), according to the table below. After 2 h incubation, aliquots of 2 µl from each sample were combined in 400 µl of 0.2% TFA, desalted, and analysed on the mass spectrometer (same method as for TMT, but without RTS) to check labelling efficiency and estimate total sample abundances. The main sample aliquots were quenched by adding 8 µl of 5% hydroxylamine, and then combined to roughly level abundances and desalted using a C18 Sep-Pak cartridge (200 mg, Waters). The eluted peptides were dissolved in 500 µl of 25 mM NH4HCO3 and fractionated by high pH reversed phase HPLC. Using an ACQUITY Arc Bio System (Waters), the samples were loaded to an XBridge® 5 µm BEH C18 130 Å column (250 x 4.6 mm, Waters). Fractionation was performed with the following gradient of solvents A (water), B (acetonitrile), and C (25 mM NH4HCO3 in water) at a flow rate of 1 ml min-1 : solvent C was kept at 10% throughout the gradient; solvent B: 0-5 min: 5%, 5-10 min: 5-10%, 10-80 min: 10- 50%, 80-90 min: 50-80%, followed by 5 min at 80% B and re-equilibration to 5% for 24 min. Fractions of 1 ml were collected and concatenated by combining fractions of similar peptide concentration to produce 24 final fractions for MS analysis. Aliquots were analysed by nanoLC-MS/MS on an Orbitrap Eclipse™ Tribrid™ mass spectrometer equipped with a FAIMS Pro Duo interphase coupled to an UltiMate® 3000 RSLCnano LC system (Thermo Fisher Scientific, Hemel Hempstead, UK). The samples were loaded onto a trap cartridge (Pepmap 100, C18, 5um, 0.3x5mm, Thermo) with 0.1% TFA at 15 µl min-1 for 3 min. The trap column was then switched in-line with the analytical column (nanoEase M/Z column, HSS C18 T3, 1.8 µm, 100 Å, 250 mm x 0.75 µm, Waters) for separation using the following gradient of solvents A (water, 0.1% formic acid) and B (80% acetonitrile, 0.1% formic acid) at a flow rate of 0.2 µl min-1 : 0-3 min 3% B (parallel to trapping); 3-10 min increase B to 8 % (curve 4); 10-108 min linear increase of B to 50%; followed by a ramp to 99% B and re-equilibration to 3% B. Data were acquired with the following parameters in positive ion mode: MS1/OT: resolution 120k, profile mode, mass range m/z 400-1600, AGC target 4e5 , max inject time 50 ms, FAIMS device set to three compensation voltages (CV: -35V, -50V, -65V) for 1 s each; MS2/IT: for each CV, data dependent analysis with the following parameters: rapid mode, centroid mode, quadrupole isolation window 0.7 Da, charge states 2-5, threshold 1.9e4 , CID CE = 30, AGC target 1.9e4, max. inject time 50 ms, dynamic exclusion 1 count for 15 s mass tolerance of 7 ppm; MS3 synchronous precursor selection (SPS): 10 SPS precursors, isolation window 0.7 Da, HCD fragmentation with CE=50, Orbitrap Turbo TMT and TMTpro resolution 30k, AGC target 200%, max inject time 100 ms, Real Time Search [2]: protein database Paeruginosa_UP000002438_208964 (uninprot.org, August 2022, 5564 entries), enzyme trypsin, 1 missed cleavage, oxidation (M) as variable, carbamidomethyl (C) and TMTpro as fixed modifications, precursor tolerance 10 ppm, Xcorr = 1.4, dCn = 0.1. The raw data were processed and quantified in Proteome Discoverer 3.1 (Thermo Fisher Scientific); all mentioned tools of the following workflow are nodes of the proprietary Proteome Discoverer (PD) software. The Paeruginosa_UP000002438_208964 protein fasta database was imported into PD adding a reversed sequence database for decoy searches; a database for common contaminants (maxquant.org, 245 entries) was also included. The database search was performed using the incorporated search engines Comet and CHIMERYS (MSAID, Munich, Germany). The processing workflow for both engines included spectrum selection and reporter ion quantification by most confident centroid (20 ppm). For CHIMERYS the Top N Peak Filter was used with 20 peaks per 100 Da the inferys_3.0.0_fragmentation prediction model was used with fragment tolerance of 0.3 Da, enzyme trypsin with 1 missed cleavage, variable modification oxidation (M), fixed modifications carbamidomethyl (C) and TMT16plex on N-terminus and K. For Comet the version 2019.01 rev. 0 parameter file was used with default settings except precursor tolerance set to 6 ppm and trypsin missed cleavages set to 1. Modifications were the same as for CHIMERYS. Evaluation of the Comet search results was performed using the Percolator node based on the q -values. Identifications were calculated for False Discovery Rate (FDR) 0.01 (strict) and 0.05 (relaxed) for both search engines. The consensus workflow in the PD software included the following parameters: assigning the 4 replicates/channels as described above (and in the table below) per condition, only unique peptides (protein groups) for quantification, intensity-based abundance, TMT channel correction values applied (WJ329096), co-isolation/SPS matches thresholds 50%/70%, normalised CHIMERYS Coefficient Threshold 0.8, normalisation on total peptide abundances, protein abundance-based ratio calculation, missing values imputation by low abundance resampling, hypothesis testing by background based t-test; the adjusted p-value is calculated in PD using the Benjamini-Hochberg method. The results were exported into a Microsoft Excel table including data for normalised and un-normalised abundances, ratios for the specified conditions, the corresponding p-values and adjusted p-values, number of unique peptides, q-values, and PEP-values from Percolator, CHIMERYS and Comet identification scores, FDR confidence combined for both search engines filtered for high confidence (strict FDR 0.01) only. Further filtering included removal of contaminants and single unique peptide matches.

TMT tag

126 WT1

127N Mut1

127C WT2

128N Mut2

128C WT3

129N Mut3

129C WT4

130N Mut4

**References:**

1. Wessel, D. and U.I. Flugge, *A method for the quantitative recovery of protein in dilute solution in the presence of detergents and lipids.* Anal Biochem, 1984. **138**(1): p. 141-3.
