# Supplementary material for: A novel stress response pathway mediates biofilm architecture in Pseudomonas aeruginosa
Source: PLoS Pathog. 2026 Jul 28;22(7):e1013832. doi: 10.1371/journal.ppat.1013832 (PMC13411936; doi:10.1371/journal.ppat.1013832)
Supplement: S5 Data — (DOCX) [file ppat.1013832.s005.docx]

**S5 data. Co-Immunoprecipitation and mass spectrometry analysis.**

Four independent cultures of PAO1 strains WT-pME-*batR* and WT-pME-3xFLAG-*batR* were grown overnight in LB medium supplemented with antibiotics and IPTG. Bacterial cells were pelleted by centrifugation, re-suspended in ice-cold IP buffer (20 mM HEPES pH 7.4, 100 mM NaCl, 1 mM EDTA, 1.0% v/v Triton X-100, protease inhibitor), and incubated at 4°C with end-over-end agitation for 6 hours. Samples were then centrifuged (15,000 x g, 20 min, 4°C), and the supernatant removed and incubated with 20 μg/ml protein-A agarose beads (4°C, end-over-end agitation, 30 min) to remove non-specifically binding proteins.

Samples were then centrifuged (3,000 x g, 1 min, 4°C) to pellet the beads, an aliquot of the supernatant was taken for analysis, and the remaining supernatant was incubated overnight with 20 μg/ml Anti-FLAG® M2 Magnetic Beads (Sigma) (4°C, end-over-end agitation). Tubes were placed in the appropriate magnetic rack and the supernatant was discarded, and the beads re-suspended in 1.0 ml ice-cold IP buffer. This wash step was repeated 5 times (only once with 0.1%triton, then 4 times with the same buffer, but without triton). The beads were then re-suspended in 50 µl of SDS sample buffer (125 mM Tris-HCl, pH:6.8, 1% SDS, 20% (v/v) glycerol, and 0.004% bromophenol blue) and heated at 95°C for 3 min. Beads were removed and the proteins were precipitated from the supernatant with chloroform/methanol. Protein pellets were resuspended in 50 µl of 1.5% sodium deoxycholate (SDC; Merck) in 0.2 M EPPS-buffer (Merck), pH 8.5 and vortexed under heating. Cysteine residues were reduced with dithiothreitol, alkylated with iodoacetamide, and the proteins digested with trypsin in the SDC buffer according to standard procedures.

Protein pellets were resuspended in 50 µl of 1.5% sodium deoxycholate (SDC; Merck) in 0.2 M EPPS-buffer (Merck), pH 8.5 and vortexed under heating. Cysteine residues were reduced with dithiothreitol, alkylated with iodoacetamide, and the proteins digested with trypsin in the SDC buffer according to standard procedures. After the digest, the SDC was precipitated by adjusting to 0.2% trifluoroacetic acid (TFA), and the clear supernatant subjected to C18 SPE using home-made stage tips with C18 Reprosil_pur 120, 5 µm (Dr Maisch, Germany). The peptides were analysed by nanoLC-MS/MS on an Orbitrap Eclipse™ Tribrid™ mass spectrometer coupled to an UltiMate® 3000 RSLCnano LC system (Thermo Fisher Scientific, Hemel Hempstead, UK). The samples were loaded onto a trap cartridge (PepMap™ Neo Trap Cartridge, C18, 5um, 0.3x5mm, Thermo) with 0.1% TFA at 15 µl min-1 for 3 min. The trap column was then switched in-line with the analytical column (Aurora Frontier TS, 60 cm nanoflow UHPLC column, ID 75 µm, reversed phase C18, 1.7 µm, 120 Å; IonOpticks, Fitzroy, Australia) for separation at 55°C using the following gradient of solvents A (water, 0.1% formic acid) and B (80% acetonitrile, 0.1% formic acid) at a flow rate of 0.26 µl min-1 : 0-3 min 0% B (parallel to trapping); 3-10 min increase B (curve 4) to 6%; 10-148 min linear increase B to 45%; followed by a ramp to 99% B and re-equilibration to 0% B, for a total of 180 min runtime. Mass spectrometry data were acquired with the FAIMS device set to three compensation voltages (-35V, -50V, -65V) at standard resolution for 1.0 s each with the following MS settings in positive ion mode: OT resolution 120K, profile mode, mass range m/z 300-1800, normalized AGC target 100%, max inject time 50 ms; MS2 in IT Turbo mode: quadrupole isolation window 1 Da, charge states 2-5, threshold 1e4, HCD CE = 30, AGC target standard, max. injection time dynamic, dynamic exclusion 1 count for 15 s with mass tolerance of ±10 ppm.

The mass spectrometry raw data were processed and quantified in Proteome Discoverer 3.0 (Thermo) using the search engine CHIMERYS (MSAID, Munich, Germany). All mentioned tools of the following workflow are nodes of the proprietary Proteome Discoverer (PD) software. The protein database Paeruginosa_UP000002438_208964 (uniprot.org, August 2022, 5563 entries) was imported into PD adding a reversed sequence database for decoy searches; databases with the special construct sequences and common contaminants (maxquant.org, 245 entries) were also included. The processing workflow included the Minora feature detector with min. trace length 7, S/N 3, PSM confidence high; the Top N Peak Filter with 10 peaks per 100 Da; Percolator with FDR targets 0.01 (strict and 0.05 (relaxed). The CHIMERYS search used the inferys_2.1_fragmentation prediction model, a fragment tolerance of 0.5 Da, enzyme trypsin with 2 missed cleavages, variable modification oxidation (M), fixed modification carbamidomethyl (C). The consensus workflow included the following parameters: normalisation on total peptide abundances, protein abundance-based ratio calculation using the top3 most abundant peptides, missing values imputation by low abundance resampling, hypothesis testing by t-test (background based), adjusted p-value calculation by BH-method. The results were exported into a Microsoft Excel table including data for normalised and un-normalised abundances, ratios for the specified conditions, the corresponding p-values and adjusted p-values, number of unique peptides, q-values and PEP-values from Percolator, CHIMERYS identification scores, FDR confidence (strict FDR 0.01 only). Further filtering included removal of contaminants and single unique peptide matches.
