# Supplementary material for: A novel stress response pathway mediates biofilm architecture in Pseudomonas aeruginosa
Source: PLoS Pathog. 2026 Jul 28;22(7):e1013832. doi: 10.1371/journal.ppat.1013832 (PMC13411936; doi:10.1371/journal.ppat.1013832)
Supplement: S6 Data — (DOCX) [file ppat.1013832.s006.docx]

**S6 data. Label-free Proteomics**

PAO1 strains were grown for 24h on LB agar plates (four biological replicates each). The cells were scraped and resuspended in 5 mL PBS buffer and further passed through a 0.2 μm syringe filter to remove bacterial cells. Filtered supernatants were used for DNA quantitation; bacteriophages assays; and analysed by label-free proteomics for protein identification. Proteins were precipitated using four volumes of acetone, mixed, and incubated for 5 minutes at room temperature. Samples were then centrifuged at high speed, and the supernatant was discarded. Protein pellets were resuspended in 50 µl of 1.5% sodium deoxycholate (SDC; Merck) in 0.2 M EPPS-buffer (Merck), pH 8.5 and resuspended by vortexing under heating. Cysteine residues were reduced with dithiothreitol, alkylated with iodoacetamide, and the proteins digested with trypsin in the SDC buffer according to standard procedures. After the digest, the SDC was precipitated by adjusting to 0.2% trifluoroacetic acid (TFA), and the clear supernatant subjected to C18 SPE using home-made stage tips with C18 membrane plugs (Supelco Analytical - 3M, Bellafonte, PA). Aliquots were analysed by nanoLC-MS/MS on an Orbitrap Eclipse™ Tribrid™ mass spectrometer equipped with a FAIMS Pro Duo interphase coupled to an UltiMate® 3000 RSLCnano LC system (Thermo Fisher Scientific, Hemel Hempstead, UK). The samples were loaded onto a trap cartridge (PepMap™ Neo Trap Cartridge, C18, 5um, 0.3x5mm, Thermo) with 0.1% TFA at 15 µl min-1 for 3 min. The trap column was then switched in-line with the analytical column (Aurora Frontier TS, 60 cm nanoflow UHPLC column, ID 75 µm, reversed phase C18, 1.7 µm, 120 Å; IonOpticks, Fitzroy, Australia) for separation at 60°C using the following gradient of solvents A (water, 0.1% formic acid) and B (80% acetonitrile, 0.1% formic acid) at a flow rate of 0.25 µl min-1 : 0-3 min 1% B (parallel to trapping); 3-10 min increase B (curve 4) to 8%; 10-102 min linear increase B to 48%; followed by a ramp to 99% B and re-equilibration to 1% B, for a total of 140 min runtime. Mass spectrometry data were acquired with the FAIMS device set to three compensation voltages (-35V, -50V, -65V) at standard resolution for 1.0 s each with the following MS settings in positive ion mode: OT resolution 120K, profile mode, mass range m/z 300-1600, normalized AGC target 100%, max inject time 50 ms; MS2 in IT Turbo mode: quadrupole isolation window 1 Da, charge states 2-5, threshold 1e4, HCD CE = 30, AGC target standard, max. injection time dynamic, dynamic exclusion 1 count for 15 s with mass tolerance of ±10 ppm.

The mass spectrometry raw data were processed and quantified in Proteome Discoverer 3.2 (Thermo) using the search engine CHIMERYS (MSAID, Munich, Germany); all mentioned tools of the following workflow are nodes of the proprietary Proteome Discoverer (PD) software. The reference proteome database for Pseudomonas aeruginosa from uniprot.org (UP000002438) was used to identify the proteins. A database for common contaminants (maxquant.org, 246 entries, Aug 2024) was also included. The databases were imported into PD adding a reversed sequence database for decoy searches. The workflow included the recalibration node (RC), the Minora Feature Detector with min. trace length 5, S/N 2.5, PSM confidence high and the TopN peak filter with 20/100 Da. The CHIMERYS database search was performed with the inferys_4.7.0_fragmentation prediction model, a fragment tolerance of 0.3 Da, enzyme trypsin with 2 missed cleavages, variable modification oxidation (M), fixed modification carbamidomethyl (C) and FDR targets 0.01 (strict) and 0.05 (relaxed). The consensus workflow in the PD software was used to evaluate the peptide identifications and to measure the abundances of the peptides based on the LC-peak intensities. For identification, an FDR of 0.01 was used as strict threshold, and 0.05 as relaxed threshold.

For quantification, 4 replicates per condition were measured. In PD3.2, the following parameters were used for ratio calculation: for chromatographic alignment and feature linking a retention time tolerance of 2 min, mass tolerance of 1 ppm and S/N 3, normalisation on total peptide abundances, protein abundance-based ratio calculation using the top3 most abundant peptides, missing values imputation by low abundance resampling, hypothesis testing by t-test (background based), adjusted p-value calculation by BH-method. The results were exported to a Microsoft Excel table including data for protein abundances, ratios, p-values, number of peptides, protein coverage, the CHIMERYS identification score and other important values.
