# Supplementary material for: A novel stress response pathway mediates biofilm architecture in Pseudomonas aeruginosa
Source: PLoS Pathog. 2026 Jul 28;22(7):e1013832. doi: 10.1371/journal.ppat.1013832 (PMC13411936; doi:10.1371/journal.ppat.1013832)
Supplement: S1 Fig — Where indicated, cultures were supplemented with tetracycline and IPTG. Mean growth from 5 biological replicates is shown as a solid line, with standard deviation indicated as dotted lines. Cells were grown for 24 h at 37 °C under shaking conditions. (DOCX) [file ppat.1013832.s007.docx]

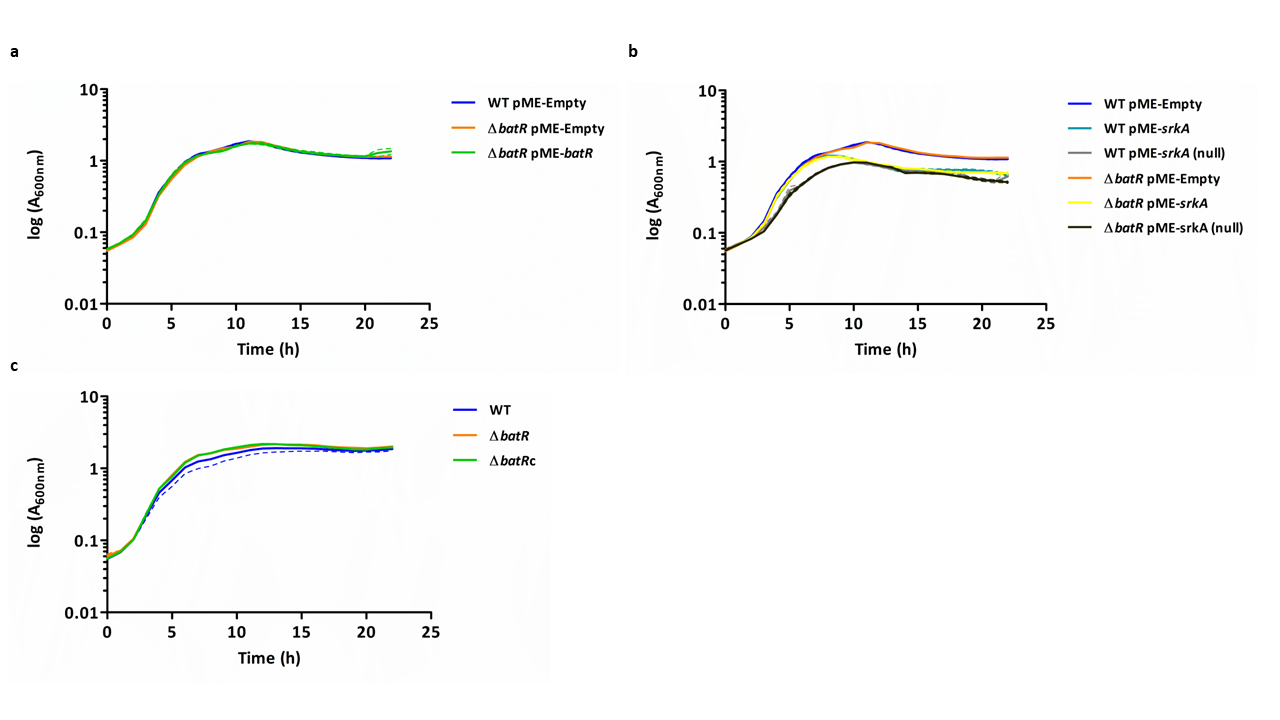


**S1 Fig.** Growth curves are shown for the following strains **a** WT pME-Empty, Δ*batR* pME-Empty and Δ*batR* pME-*batR*; **b** WT pME-Empty, Δ*batR* pME-Empty, WT pME-*srkA*, Δ*batR* pME-*srkA,* WT pME-*srkA* (null) and Δ*batR* pME-*srkA* (null); and **c** WT, Δ*batR* and Δ*batR*c grown in LB medium. Where indicated, cultures were supplemented with tetracycline and IPTG. Mean growth from 5 biological replicates is shown as a solid line, with standard deviation indicated as dotted lines. Cells were grown for 24 h at 37 °C under shaking conditions.
