# Supplementary material for: A novel stress response pathway mediates biofilm architecture in Pseudomonas aeruginosa
Source: PLoS Pathog. 2026 Jul 28;22(7):e1013832. doi: 10.1371/journal.ppat.1013832 (PMC13411936; doi:10.1371/journal.ppat.1013832)
Supplement: S2 Fig — Drug accumulation was assessed by measuring resazurin fluorescence at an excitation wavelength of 544 nm and an emission wavelength of 590 nm (544/590 nm) for 2 hours (a), 12 hours (b) and 18 hours (c). Lines represent the mean of three biological replicates, each with four technical replicates, with the error bars showing the standard deviation. No significant differences were observed between the strains tested. (DOCX) [file ppat.1013832.s008.docx]

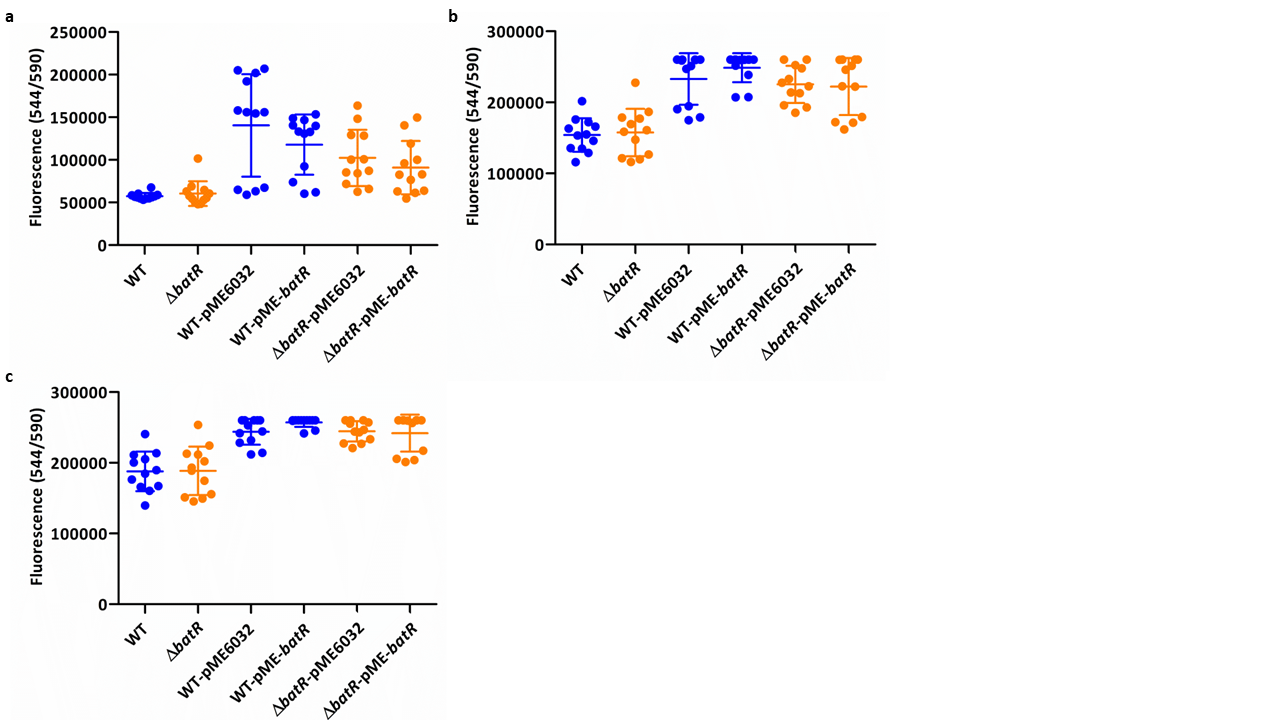


**S2 Fig. BatR does not affect membrane permeability.** Drug accumulation was assessed by measuring resazurin fluorescence at an excitation wavelength of 544 nm and an emission wavelength of 590 nm (544/590 nm) for 2 hours (a), 12 hours (b) and 18 hours (c). Lines represent the mean of three biological replicates, each with four technical replicates, with the error bars showing the standard deviation. No significant differences were observed between the strains tested.
