# Supplementary material for: A novel stress response pathway mediates biofilm architecture in Pseudomonas aeruginosa
Source: PLoS Pathog. 2026 Jul 28;22(7):e1013832. doi: 10.1371/journal.ppat.1013832 (PMC13411936; doi:10.1371/journal.ppat.1013832)
Supplement: S3 Fig — Genetic organization of the R2 pyocin gene locus in P. aeruginosa PAO1. Structure of the R2-F2 pyocin gene cluster in P. aeruginosa PAO1. Grey indicates regulatory genes; yellow, the lysis cassette; green, R2-type pyocin genes; and orange, F2-type pyocin genes. The function of individual proteins is labelled, and proteins enriched in the WT strain proteome are boxed. Gene sizes are drawn to scale. b. Effect of BatR on pyocin production. The effect of BatR on the promoter activity of PA0614 was measured using pME0614-G, normalised to pMEXGFP. c. Proportion of cells exhibiting a round-cell morphotype in microfluidics assays. Cells were classified as either rod-shaped or round over a 3-hour period, and the frequency of round cells was calculated as the number of round cells divided by the total number of cells counted at t = 180 min. Results were analysed by a one-way ANOVA showing no significant differences between WT and ΔbatR strains under the conditions tested. d. Phase-contrast time-lapse images showing rod-to-round cell transition and explosive lysis events (highlighted by red circles) in WT and ΔbatR strains. Time is indicated in minutes (top right); scale bar, 2 μm. (DOCX) [file ppat.1013832.s009.docx]

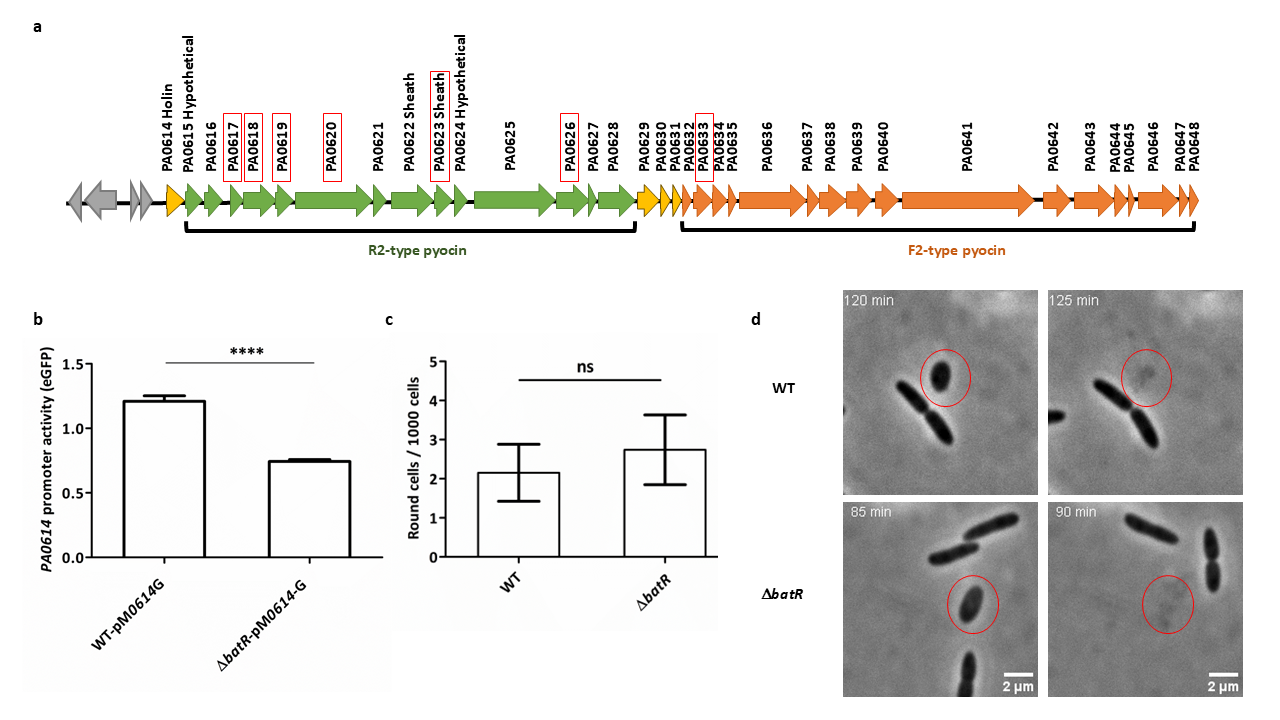


**S3 Fig. a. Genetic organization of the R2 pyocin gene locus in *P. aeruginosa* PAO1.** Structure of the R2-F2 pyocin gene cluster in *P. aeruginosa* PAO1. Grey indicates regulatory genes; yellow, the lysis cassette; green, R2-type pyocin genes; and orange, F2-type pyocin genes. The function of individual proteins is labelled, and proteins enriched in the WT strain proteome are boxed. Gene sizes are drawn to scale. **b.** **Effect of BatR on pyocin production.** The effect of BatR on the promoter activity of *PA0614* was measured using pME0614-G, normalised to pMEXGFP. **c. Proportion of cells exhibiting a round-cell morphotype in microfluidics assays.** Cells were classified as either rod-shaped or round over a 3-hour period, and the frequency of round cells was calculated as the number of round cells divided by the total number of cells counted at *t* = 180 min. Results were analysed by a one-way ANOVA showing no significant differences between WT and Δ*batR* strains under the conditions tested. **d.** Phase-contrast time-lapse images showing rod-to-round cell transition and explosive lysis events (highlighted by red circles) in WT and Δ*batR* strains. Time is indicated in minutes (top right); scale bar, 2 μm.
