# Supplementary material for: A novel stress response pathway mediates biofilm architecture in Pseudomonas aeruginosa
Source: PLoS Pathog. 2026 Jul 28;22(7):e1013832. doi: 10.1371/journal.ppat.1013832 (PMC13411936; doi:10.1371/journal.ppat.1013832)
Supplement: S4 Fig — a. Biofilm formation. Strains were grown statically in LB medium for 24h at 37°C. Biofilm biomass was quantified by Crystal Violet staining and measured spectrophotometrically at 590 nm (A590nm.). Values represent the mean of five biological replicates with two technical replicates each; error bars indicate SD. b. Cell viability. Cells were scraped from LB agar plates, resuspended in PBS, and enumerated via serial dilution and plating. c. Pyocyanin production. Top view images of cell lawns showing pyocyanin production (blue colouration). d. Growth curve. Growth curves are shown for strains WT (blue), ΔbatR (orange), ΔsrkA (gray); and ΔbatR ΔsrkA (yellow)in LB medium. The mean growth for 6 biological replicates is shown as a solid line and standard deviation shown as dotted lines. Cells were grown for 48 h at 37 °C under shaking conditions. e, f. Influence of batR and srkA deletions on PA0629 (lys) and PA0727 (Pf4) transcripts, respectively as determined by qRT-PCR. Transcript levels were normalised against rpoD. One-way ANOVA with Tukey's multiple comparisons was used to compare means, p < 0.0001 ***. (DOCX) [file ppat.1013832.s010.docx]

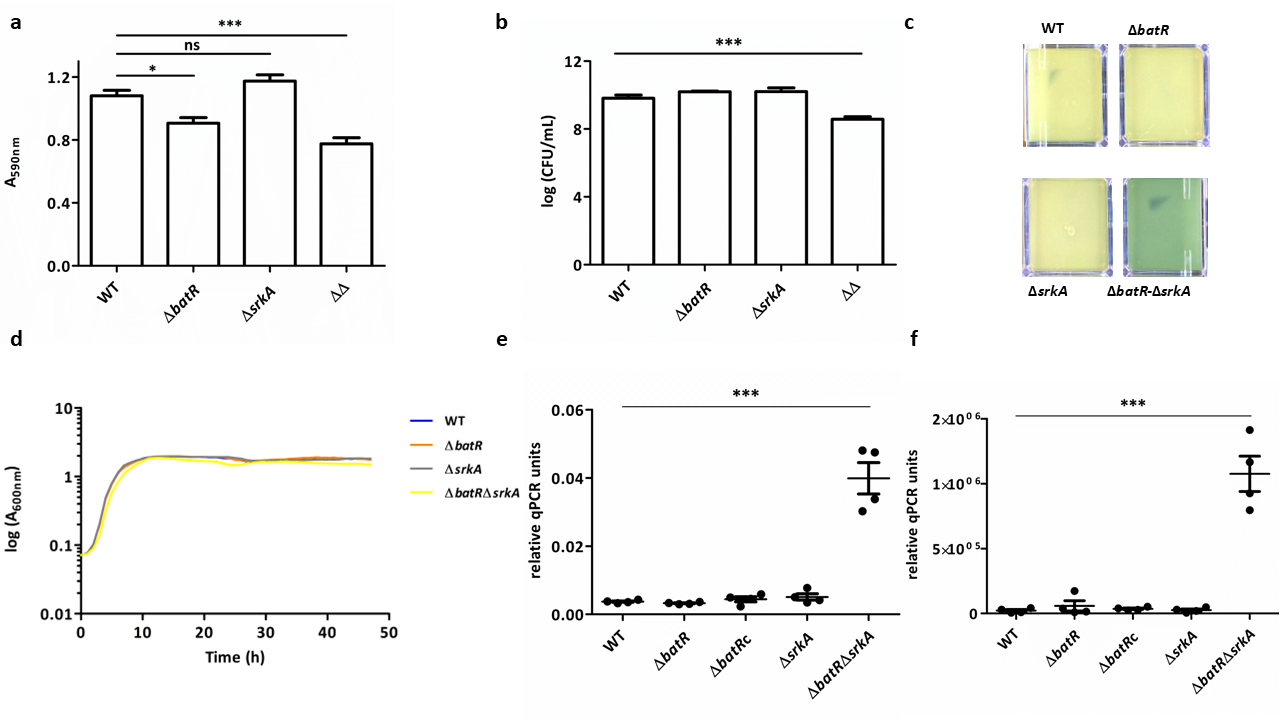


**S4 Fig. Phenotypes of PAO1 WT; ∆*batR;* ∆*srkA;* and ∆*batR* ∆*srkA* strains.** **a. Biofilm formation.** Strains were grown statically in LB medium for 24h at 37°C. Biofilm biomass was quantified by Crystal Violet staining and measured spectrophotometrically at 590 nm (A_590nm_.). Values represent the mean of five biological replicates with two technical replicates each; error bars indicate SD. **b. Cell viability.** Cells were scraped from LB agar plates, resuspended in PBS, and enumerated via serial dilution and plating. **c. Pyocyanin production.** Top view images of cell lawns showing pyocyanin production (blue colouration). **d. Growth curve.** Growth curves are shown for strains WT (blue), Δ*batR* (orange), ΔsrkA (gray); and Δ*batR* ΔsrkA (yellow)in LB medium. The mean growth for 6 biological replicates is shown as a solid line and standard deviation shown as dotted lines. Cells were grown for 48 h at 37 °C under shaking conditions. **e, f.** Influence of *batR* and *srkA* deletions on *PA0629* (*lys*) and *PA0727* (*Pf4*) transcripts, respectively as determined by qRT-PCR. Transcript levels were normalised against *rpoD*. One-way ANOVA with Tukey's multiple comparisons was used to compare means, p<0.0001 ***.
