# Supplementary material for: A novel stress response pathway mediates biofilm architecture in Pseudomonas aeruginosa
Source: PLoS Pathog. 2026 Jul 28;22(7):e1013832. doi: 10.1371/journal.ppat.1013832 (PMC13411936; doi:10.1371/journal.ppat.1013832)
Supplement: S5 Fig — The depth graph shows the distribution of sequencing read coverage across the genomic region spanning the Pf4 cluster (PA0717-PA0727), with each gene represented by a blue arrow. Three PAO1 WT-derived mutants overexpressing srkA null are shown. (DOCX) [file ppat.1013832.s011.docx]

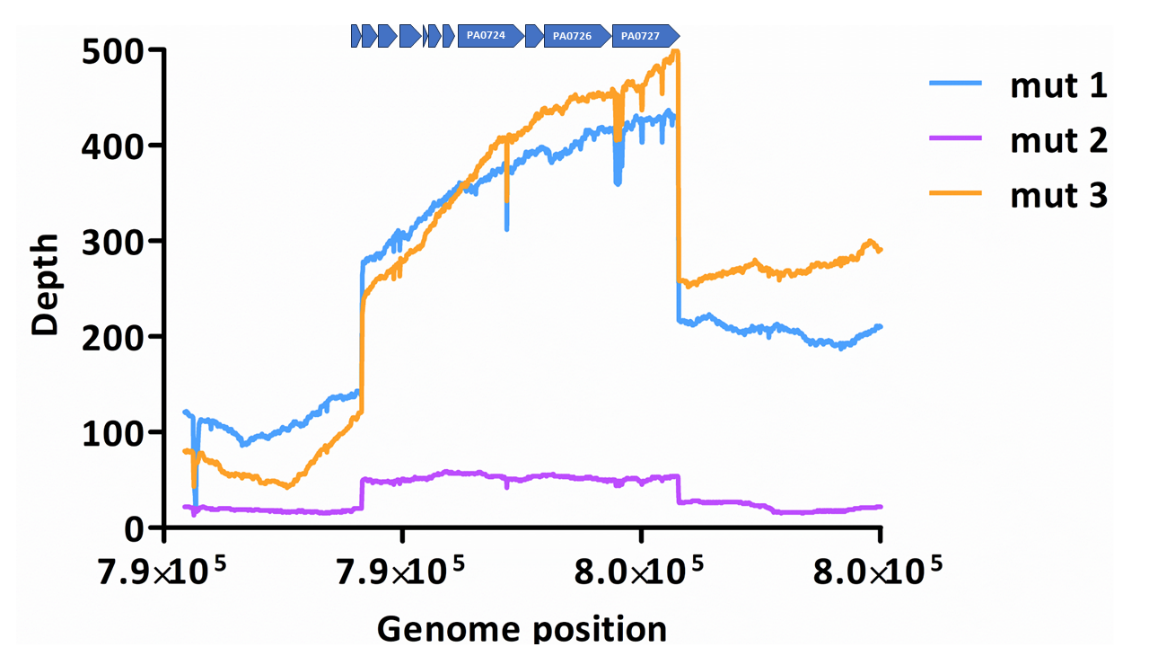


**S5 Fig. Overexpression of *srkA* null activates prophage Pf4 in *P. aeruginosa*.** The depth graph shows the distribution of sequencing read coverage across the genomic region spanning the Pf4 cluster (PA0717-PA0727), with each gene represented by a blue arrow. Three PAO1 WT-derived mutants overexpressing *srkA* null are shown.
