# Supplementary material for: A novel stress response pathway mediates biofilm architecture in Pseudomonas aeruginosa
Source: PLoS Pathog. 2026 Jul 28;22(7):e1013832. doi: 10.1371/journal.ppat.1013832 (PMC13411936; doi:10.1371/journal.ppat.1013832)
Supplement: S6 Fig — Log-transformed total CFU of P. aeruginosa PAO1 WT recovered from the EVPL model following treatment with different concentrations of CIP. Three biological replicates were grown on EVPL tissue for 48 h, then exposed to CIP or PBS as a control for 18 h. CFU/lung was determined post treatment. The MIC of CIP in this model was 128 µg/mL. (DOCX) [file ppat.1013832.s012.docx]

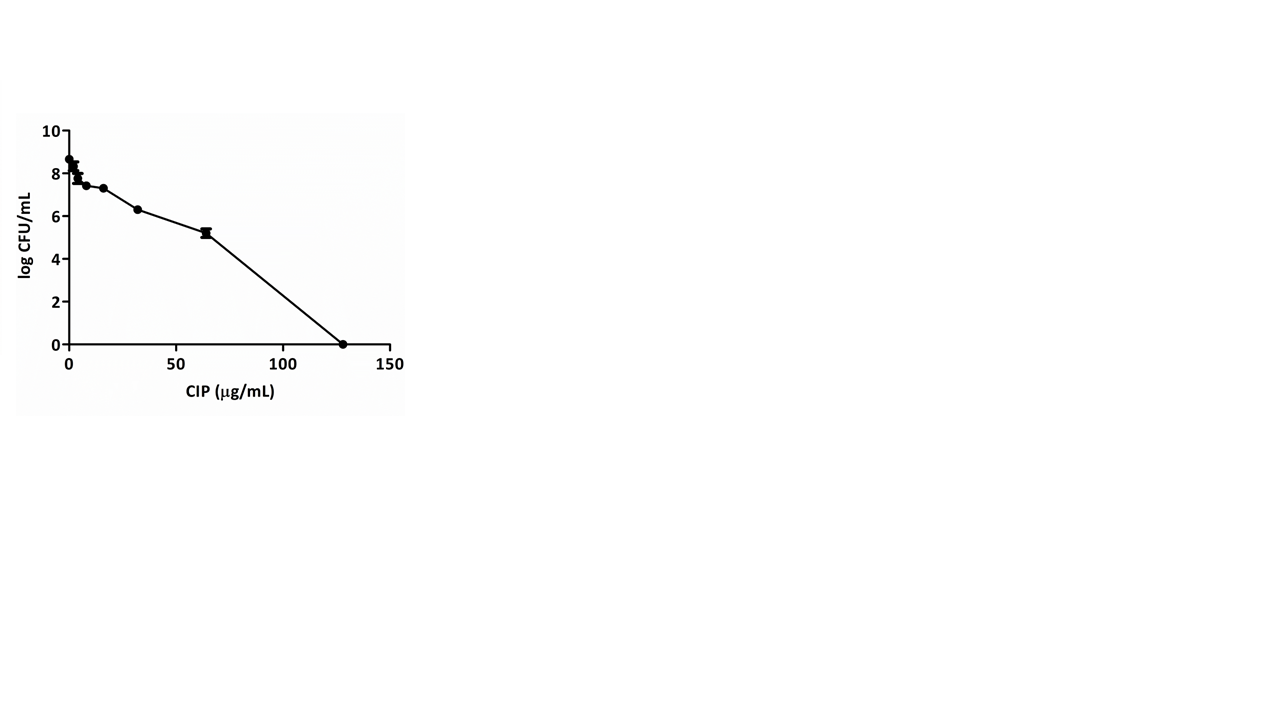


**S6 Fig. MIC of PAO1 wild type in the EVPL Model**. Log-transformed total CFU of *P. aeruginosa* PAO1 WT recovered from the EVPL model following treatment with different concentrations of CIP. Three biological replicates were grown on EVPL tissue for 48 h, then exposed to CIP or PBS as a control for 18 h. CFU/lung was determined post treatment. The MIC of CIP in this model was 128 µg/mL.
