# Supplementary material for: A novel stress response pathway mediates biofilm architecture in Pseudomonas aeruginosa
Source: PLoS Pathog. 2026 Jul 28;22(7):e1013832. doi: 10.1371/journal.ppat.1013832 (PMC13411936; doi:10.1371/journal.ppat.1013832)
Supplement: S7 Fig — EVPL was infected with P. aeruginosa PAO1 WT and ∆batR, with uninfected tissue as a negative control. The x20 magnification images from the sections are shown here for non-treated tissues (SCFM) and treated with CIP (SCFM + CIP). Representative images of phenotypes at 2dpi are shown here, but the same results were observed for all biological replicates analysed. (DOCX) [file ppat.1013832.s013.docx]

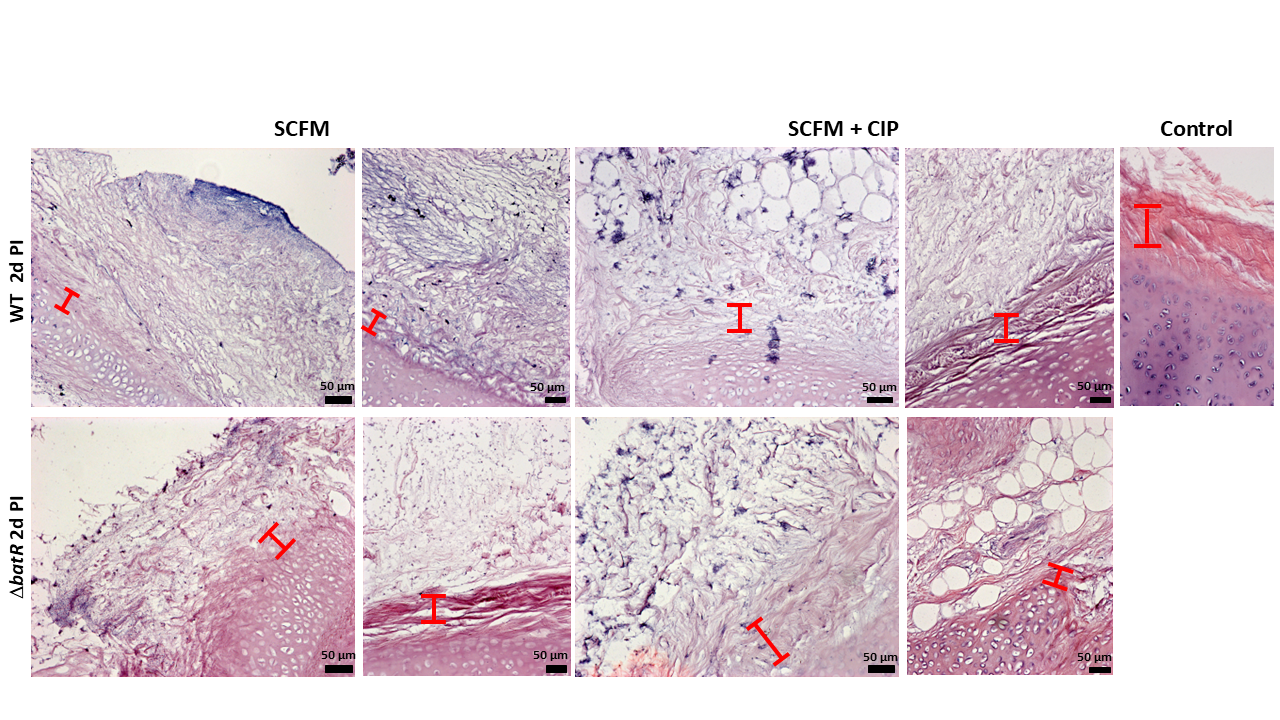


**S7 Fig. Haematoxylin and eosin (H & E) stained sections of EVPL bronchiolar tissue with SCFM medium infected with *P. aeruginosa* at 2 d post infection.** EVPL was infected with *P. aeruginosa* PAO1 WT and ∆*batR*, with uninfected tissue as a negative control. The x20 magnification images from the sections are shown here for non-treated tissues (SCFM) and treated with CIP (SCFM + CIP). Representative images of phenotypes at 2dpi are shown here, but the same results were observed for all biological replicates analysed.
